# The New Era of High-Throughput Nanoelectrochemistry

**DOI:** 10.1021/acs.analchem.2c05105

**Published:** 2023-01-10

**Authors:** Xiangdong Xu, Dimitrios Valavanis, Paolo Ciocci, Samuel Confederat, Fabio Marcuccio, Jean-François Lemineur, Paolo Actis, Frédéric Kanoufi, Patrick R. Unwin

**Affiliations:** † Department of Chemistry, University of Warwick, Coventry CV4 7AL, U.K.; ‡ Université Paris Cité, ITODYS, CNRS, F-75013 Paris, France; § School of Electronic and Electrical Engineering and Pollard Institute, University of Leeds, Leeds LS2 9JT, U.K.; ∥ Bragg Centre for Materials Research, University of Leeds, Leeds LS2 9JT, U.K.; ⊥ Faculty of Medicine, Imperial College London, London SW7 2AZ, United Kingdom

To acquire a deeper understanding of electrochemical systems requires techniques that address nanoscale aspects of electrochemistry, *inter alia*, the detection and analysis of single entities, spatial heterogeneities in electrochemical processes and the properties of an electrochemical interface, and the role of the electrical double layer (EDL).^1^ Unveiling the complex spatiotemporal dynamics of electrochemical interfaces is the cornerstone to answering many fundamental and practical questions that are directly relevant to major societal challenges, including the development of electrochemical energy storage and conversion systems to drive the transition to low-carbon energy, and improved technologies to combat corrosion.^2^ Underpinned by fundamental understanding, especially of nanoscale phenomena, electrochemistry also offers exciting possibilities for chemical synthesis,^3^ for the creation of advanced nanomaterials, and for precise surface functionalization. Furthermore, an increasing number of sensing and diagnostic technologies make use of electrochemistry, and interfacial charge transfer is at the heart of living systems, with renewed recognition of the importance of local bioelectrical phenomena in cell biology.^4^ Finally, since electrochemistry deals with mass transport and reactions at interfaces, the subject provides a framework for addressing and describing interfacial processes generally.

Scanning electrochemical probe microscopies (SEPMs) have played a pivotal role in advancing small-scale electrochemistry. SEPMs use an electrochemical probe (micro/nanoelectrode or pipet) to quantify and map local interfacial fluxes of electroactive species and have found increasingly wide applications. Our contribution to the Fundamental and Applied Reviews in Analytical Chemistry 2019 (ref (5)) discussed how advances in SEPMs converged toward nanoscale electrochemical mapping. This inflection in experimental capability has opened up myriad opportunities for SEPMs in many types of systems, from material and energy sciences to the life sciences. The enhancement in the spatial resolution of imaging techniques, and instrumental developments, have resulted in significant increases in the size of electrochemical data sets from typical experiments and served to speed up measurement throughput. Next-generation nanoelectrochemistry will thus see an emphasis on “big data”; its analysis, storage, and curation; high-throughput analysis and parallelization; “intelligent” instruments and experiments; active control of nanoscale systems; and the integration of nanoelectrochemistry and nanoscale micro(spectro)scopy.^6^ We are witnessing radical changes to the way in which electrochemists perform and analyze experiments; this review article thus focuses on recent advances in frontier nanoscale electrochemistry and imaging techniques that address many of these key targets and are well-placed to embrace other aspects in the near future. Our goal is to provide an overview of the present state-of-the-art in high-throughput nanoelectrochemistry and microscopy and signpost promising new avenues for nanoscale electrochemical methods.

In selecting material for inclusion in this article, we consider distinct, but related areas, spanning nanopores/pipettes, nanopipette probe microscopy, and widefield imaging. These methods are becoming a natural choice for high-throughput single-entity nanoelectrochemistry,^7−9^ where the goal is to detect and analyze, *inter alia*, single molecules, single cells, and individual particles (and other nanoobjects), as well as to break down the response of complex electrode surfaces and interfaces into a set of simpler elementary features, e.g., terraces, step edges, grain boundaries, etc. Each of the areas we have selected is benefiting from similar developments in experimental capability and analysis tools; there are also efforts to integrate techniques and ideas from each of these areas. Furthermore, there is considerable overlap in the types of processes and phenomena that are studied with these different techniques, and so bringing together a discussion and comparison of the methodology is beneficial. We consider:

(i) Nanopores that are used in a wide range of fields spanning biophysics, bioanalytical sensors, and water desalination applications. They exploit the high-throughput and selective transport and screening of chemical species offered by the electric field and molecular confinement in nanopores and nanopipettes. While scholarly reviews of nanopore electrochemistry have been published recently,^10−13^ we highlight here the present state-of-the art and the importance of nanopores as a platform for high-throughput electroanalysis. Electrochemical nanoimpacts is another platform providing high-throughput electroanalysis at the level of individual nanoparticles (NPs). This methodology allows NP counting and characterization by recording at high throughput the electrochemical signature associated with a series of NP collisions, on a miniaturized electrode. The technique has allowed exploration of a wealth of electrochemical or physical (transport, electrostatics, *inter alia*) phenomena at the nanoscale. However, the potential of the methodology to resolve structure–activity is much less explored and difficult to achieve, unless the methodology is enhanced with integrated *operando* microscopy. Thus, electrochemical nanoimpacts will be discussed in parts of this article, but not addressed in a specific section, especially since recent research efforts in this area have been exhaustively summarized in authoritative reviews.^14−17^
(ii) The use of nanopipettes as local electrochemical probes in scanning ion conductance microscopy (SICM)^18^ and scanning electrochemical cell microscopy (SECCM).^19^ These two scanned probe techniques share the same type of easy-to-make and characterize probes that are readily deployed at the nanoscale (10s nm sized probes). Scanning electrochemical microscopy (SECM) is not addressed in this review, as high-throughput nanoscale resolution imaging by SECM is still rare and challenging to implement. Moreover, the third edition of the classic text on SECM, providing a comprehensive review of the field, has recently been assembled by authorities in the field.^20^
(iii) Advanced optical microscopy techniques that have increasingly been used for imaging electrochemical interfaces *operando* with nanoscale resolution, using superlocalization principles. They are now expanding to a wide range of electrochemical systems, as illustrated by several recent reviews.^21−26^ Their easy hyphenation with complementary electrochemical or structural analyses make them particularly compelling tools for high-throughput characterization as we discuss herein.

## High-Throughput Nanopores

### Nanopore Working Principle

**Figure 1:**
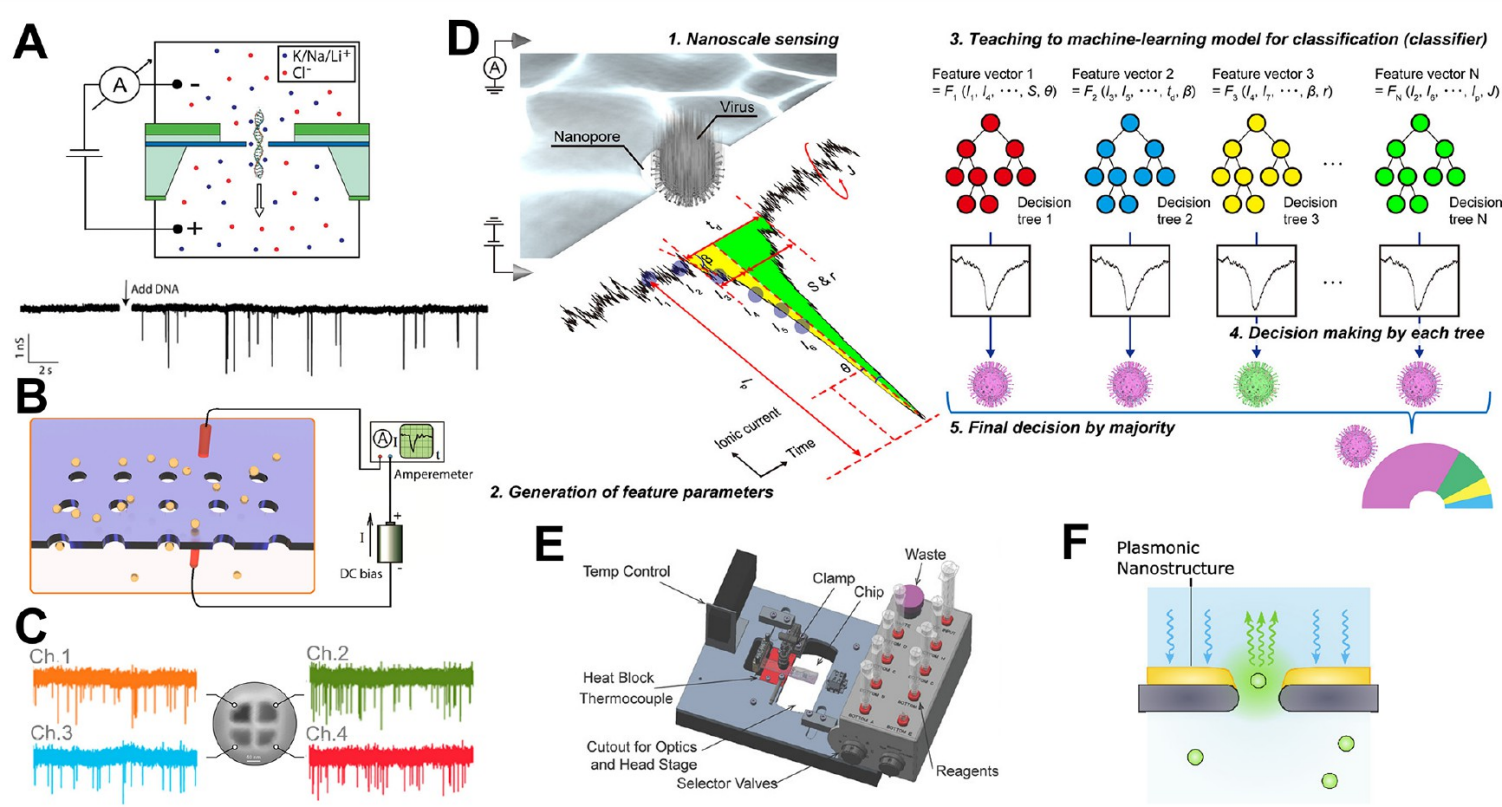
(A) Principle of nanopore sensing. A nanoscale size pore separates two reservoirs filled with electrolyte solutions. Electrodes are placed in each reservoir and a constant voltage bias is applied across the nanopore causing a charged molecule (e.g., DNA molecule) to translocate through the nanopore. Reproduced from Kowalczyk, S. W.; Wells, D. B.; Aksimentiev, A.; Dekker, C*. Nano Lett*. **2012**, *12* (2), 1038–1044 (ref (33)). Copyright 2012 American Chemical Society. (B) Schematic of a nanopore array setup used for addressing translocation of nanoparticles through multiple nanopores. Reproduced from Wen, C.; Zeng, S.; Zhang, Z.; Zhang, S. L. *Anal. Chem.***2018**, *90* (22), 13483–13490 (ref (53)). Copyright 2018 American Chemical Society. (C) SEM image of quad-barrel nanopipette depicting ionic current traces from each barrel used for the translocation of 10 kbp DNA molecules from inside the barrel into the bath. Reproduced from Cadinu, P.; Kang, M.; Nadappuram, B. P.; Ivanov, A. P.; Edel, J. B. *Nano Lett.***2020**, *20* (3), 2012–2019 (ref (58)). Copyright 2020 American Chemical Society. (D) Example of machine-learning assisted nanopore readout workflow for identification of virus particles. Reproduced from Arima, A.; Tsutsui, M.; Washio, T.; Baba, Y.; Kawai, T. *Anal. Chem*. **2021**, *93* (1), 215–227 (ref (62)). Copyright 2021 American Chemical Society. (E) Custom design of a stand-alone microfluidic device with integrated solid-state nanopore. The device compromises of multiple components to streamline fluidic, temperature, and electronic control. Reproduced from A Solid-State Hard Microfluidic-Nanopore Biosensor with Multilayer Fluidics and On-Chip Bioassay/Purification Chamber, Varongchayakul, N.; Hersey, J.; Squires, A.; Meller, A.; Grinstaff, M. *Adv. Funct. Mater.*, Vol. 28, Issue 50 (ref (63)). Copyright 2018 Wiley. (F) Schematic depicting a solid-state nanopore with an integrated plasmonic nanostructure for optical signal enhancement. Reproduced from Fried, J. P.; Wu, Y.; Tilley, R. D.; Gooding, J. J. *Nano Lett.***2022**, *22* (3), 869–880 (ref (10)). Copyright 2022 American Chemical Society.

The development of nanopore technology in the last decades has enabled numerous applications in single-entity research.^27,28^ The general nanopore readout relies on resistive pulse sensing, where the nanopore links two compartments filled with an electrolyte solution with electrodes immersed in each compartment. Upon the application of a constant voltage, analytes are translocated through the nanopore causing a temporary modulation in the measured ionic current. The characteristic of the current perturbations, such as current magnitude, duration, and frequency, provide information about the physicochemical properties of the analyte (i.e., size, charge, shape, concentration) and its interactions with the nanopore (Figure 1A).^29−31^ This sensing approach has enabled, *inter alia*, the single-molecule analysis of RNA,^32^ DNA nanostructures,^33−38^ proteins,^39−43^ ribosomes,^44^ and virus particles.^45,46^ Furthermore, nanopores and related nanopipettes (*vide infra*) have been employed in the analysis of inorganic colloids and nanoparticles.^47−50^ The most successful application of nanopore technology, however, is in the field of nucleic acid sequencing, as demonstrated by the commercialization of nanopore sequencing devices from Oxford Nanopore Technologies Ltd.^51^ This technology relies on the integration of biological nanopores with advanced electronics, and some recent applications of biological nanopores were reviewed recently.^11,52^

In this section, we highlight recent work enabling high-throughput single-entity detection with nanopores and implications for single-entity research. We present key aspects to allow for high-throughput analysis and discuss the integration of nanopores with microfluidic/optical setups, and the application of machine-learning algorithms to process nanopore data and to enable high-resolution single-entity analysis.

### Arrayed Nanopore Configurations

One approach to increase the throughput of nanopore-based sensors is the development of parallelized measurements employing arrayed nanopore devices. Recent developments in micro/nanofabrication technologies have resulted in several fabrication methods for the production of solid-state nanopores, e.g., photolithography, transmission electron microscopy (TEM) drilling, and controlled dielectric breakdown.^30^ These methods have brought great flexibility in tuning the size and geometry of nanopores using a range of substrate materials, e.g., SiN_*x*_, SiO_2_, graphene, polymers, and glass capillaries.^30^ Capitalizing on this fabrication technology, nanopore devices can be scaled-up to produce multiple nanopores integrated in one device or the fabrication of nanopore arrays. Wen et al. employed 30–100 nanopores in a suspended SiN_*x*_ membrane for the detection of nanoparticles (Figure 1B).^53^ Similar strategies have also been reported with SiN_*x*_ and graphene-based membranes fabricated by electron-beam lithography and reactive ion etching.^54,55^ While these high-end fabrication approaches are attractive in terms of their precision, they pose challenges for large-scale production. An alternative strategy with potential for high yield and simple fabrication is the use of nanoimprint lithography with a Si microneedle stamp, as demonstrated by Choi et al.^55^ Here, sub-10 nm nanopores were fabricated in a freestanding polymer membrane in a single-step process. Also, the use of controlled dielectric breakdown serves as a versatile approach to fabricate multiple nanopores *in situ*.^56^ An attractive aspect in this case is the direct fabrication of nanopores in microfluidic devices, which can greatly reduce the numbers of fabrication and assembly steps involved in the production process.^57^ An alternative approach relies on multibarreled glass nanopipettes that can be independently controlled (Figure 1C),^58,59^ allowing for trapping and dynamic manipulation of individual molecules.

With the expansion of arrayed nanopore configurations, the requirement for parallelization of the measurement set up becomes evident^60^ and modern CMOS (complementary metal-oxide-semiconductor field-effect transistor) fabrication processes can favor new emerging strategies for the fabrication of arrayed nanopores integrated with the electronics readout.^61^

### Machine-Learning Assisted Nanopore Readout

An important aspect in expanding the high-throughput capabilities of nanopore platforms is the implementation of novel data analysis strategies and machine-learning algorithms. Ionic current characteristics are routinely used in nanopore measurements to extract physical information about the analytes investigated.^64^ While the complexity of samples investigated by nanopore-based sensors is increasing at a rapid pace, so too is the nanopore measurement throughput. New strategies are therefore needed for real-time analysis and to explore new discriminants for the detection and characterization of complex analyte mixtures. In order to classify each signal, various features are extracted from the current–time waveforms recorded. Conventionally, the peak height and the width of the signal are used to discriminate molecules during nanopore translocation.^64^ However, additional features can be used to enhance signal classification, such as the peak area, rise and decay time, frequency of events, and subpeak levels, to name just a few.^62^ Such features can serve as the input for machine learning algorithms to enhance the readout efficiency and lay the ground for enhanced analyte identification and classification, as exemplified in Figure 1D.

Several studies on nanopore-based sensing have demonstrated the implementation of postprocessing machine learning algorithms for quantitative and qualitative applications. The general approach for the development of machine learning algorithms is to build a training process, including several stages such as data importing, extraction of defined features, model training, and evaluation of the model.^65^ Hu et al. demonstrated the use of a machine learning algorithm to obtain automated classification of metabolites using a nanopore platform.^66^ Taniguchi et al. reported the identification of several polystyrene nanoparticles by combining solid-state nanopore sensing with a machine learning algorithm.^67^ The same group has demonstrated that such approach can also be employed for accurate detection of different types of viruses.^68^ A further example of a machine learning approach for discriminating analytes in mixtures is the use of the learning time-series shapelets, as exemplified by Wei et al.^69^ Here, the maximum discriminative features corresponding to each analyte that form a short segment of time-series data are extracted as shapelet signals and use the shapelet-transformed representation in order to classify the analytes investigated.

A key aspect in improving the readout throughput of nanopores is enabling real-time analysis and classification readout efficiency. Moreover, synergetic efforts to define servers with commonly used databases can contribute greatly to the advancement of nanopore data analysis. Complementing ionic current signals with information obtained from other complementary techniques (i.e., fluorescence and Raman techniques) would generate a platform with high-throughput readout for single-molecule investigations.

### Integration Modalities for Nanopores

The integration of nanopores with lab-on-chip devices can improve automation in terms of sample preparation and preconcentration, while allowing for continuous measurements. Several groups have reported the integration of nanopores within microfluidic architectures for high-throughput detection of nanoparticles.^70−72^ Varongchayakul et al. presented a standalone microfluidic-nanopore setup that supports on-chip sample preparation, purification, and single-molecule nanopore measurements (Figure 1E).^63^ With the recent development in 3D-printing technology, nanopore supports and microdevices with custom features can now be tailored in a rapid and facile manner,^73^ favoring their integration in miniaturized setups. Roman et al. reported a PDMS-based microfluidic device with an integrated nanopore utilizing 3D-printed molds.^74^

The small footprint of nanopores and the possibility to integrate them with complementary nanostructures allows their interfacing with nonelectrical readouts for increased throughput. Verschueren et al. proposed a setup comprising an inverted-bowtie plasmonic nanopore to achieve both ionic current and transmitted light detection of DNA translocation.^75^ A similar concept for combined measurement modalities was demonstrated by employing a programmable opto-fluidic chip for high-throughput single-molecule analysis.^76^ The integration of optical nanopores can potentially increase the statistics obtained with independent readouts from multiple nanopores within the field-of-view. Optical nanopore sensing strategies, such as confocal microscopy, total internal reflection fluorescence microscopy, zero mode waveguide, or plasmonic enhancement, can be explored for integration with high-density nanopore arrays and pave the way for research tools with high-throughput single-molecule detection (Figure 1F).^10^ Lastly, solid-state nanopores based on glass nanopipettes have the potential for enhanced throughput measurements due to their integration with manipulators, in an analogous way to liquid handling robots,^77^ and with scanning probe microscopy (SPM) techniques.^29^

### Nanopore-Confined Electrochemistry

**Figure 2:**
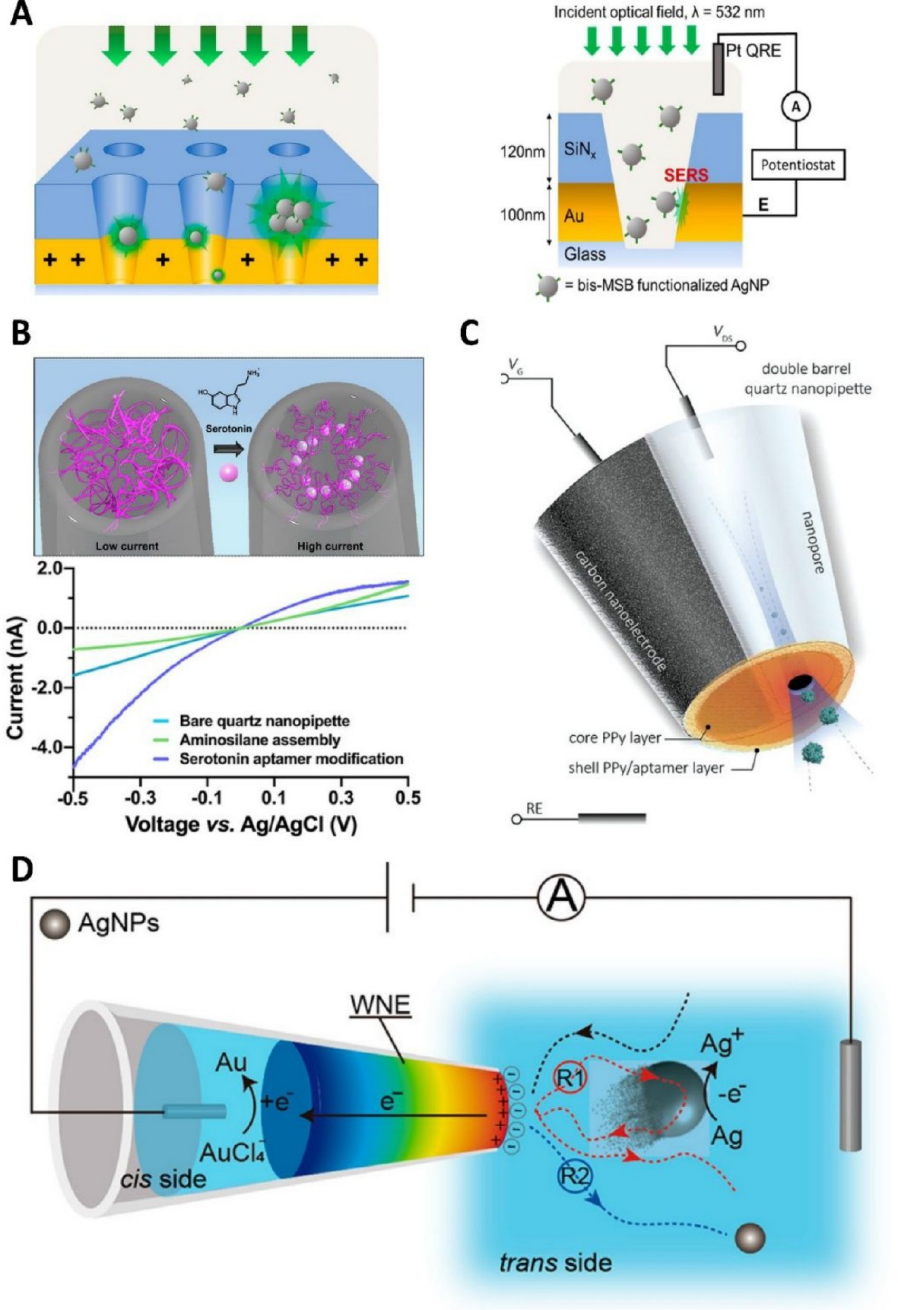
(A) Schematic representation of a high-density nanopore-electrode array and cross section of an individual nanopore configuration with functionalized silver particles captured by applying a voltage at a gold-ring electrode present within the confined pore vs a Pt quasi-reference counter electrode (QRCE). Simultaneously, SERS measurements are conducted by illuminating from the top with 532 nm incident light. Reproduced from Kim, J. Y.; Han, D.; Crouch, G. M.; Kwon, S. R.; Bohn, P. W. *Anal. Chem.***2019**, *91* (7), 4568–4576 (ref (82)). Copyright 2019 American Chemical Society. (B) Schematic depiction of ICR sensing of serotonin with aptamer-functionalized nanopipettes. Upon binding of serotonin, the aptamers undergo a conformational rearrangement that leads to a change in the ionic flux through the nanopipette, altering the ICR. Reproduced from Nakatsuka, N.; Failletaz, A.; Eggemann, D.; Forro, C.; Voros, J.; Momotenko, D. *Anal. Chem*. **2021**, *93* (8), 4033–4041 (ref (85)). Copyright 2021 American Chemical Society. (C) Schematic of a double-barrel nanopipette utilized as an extended field-effect transistor sensor for selective detection and controlled protein transport. Reproduced from Selective Sensing of Proteins Using Aptamer Functionalized Nanopore Extended Field-Effect Transistors, Ren, R.; Wang, X.; Cai, S.; Zhang, Y.; Korchev, Y.; Ivanov, A. P.; Edel, J. B. *Small Methods***2020**, Vol. 4, Issue 11 (ref (91)) under CC-BY 4.0 license. (D) Example representation of a wireless nanopore electrode utilized for nanoconfined electrochemical sensing of silver nanoparticles collisions, supporting simultaneous Faradaic and capacitive responses. Reproduced from Yu, R. J.; Xu, S. W.; Paul, S.; Ying, Y. L.; Cui, L. F.; Daiguji, H.; Hsu, W. L.; Long, Y. T. *ACS Sens.***2021**, *6* (2), 335–339 (ref (93)). Copyright 2021 American Chemical Society.

Novel concepts have emerged for electrochemistry confinement utilizing modified nanopores. Bohn and co-workers proposed high-density nanopore electrode arrays with attoliter volumes and polarizable metallic substrates to investigate nanoparticle transport and *in situ* redox reactions, isolating the nanoparticle behavior from the bulk colloidal motion.^78−81^ Furthermore, by monitoring SERS in conjunction with amperometry, enhanced information about the transport and capture of single nanoparticles can be obtained, as exemplified in Figure 2A.^82^ The asymmetric geometry of the conical nanopipette and its surface charge generates distinctive electrochemical properties enhanced by the nanoconfinement. The asymmetric ion transport under diluted electrolyte solution gives rise to the ion current rectification (ICR) effect.^83,84^ This effect has be further developed into a nanopore sensing approach where the characteristic current–voltage curves can be modulated by the changes on the surface of the nanopore induced by the presence of analytes (Figure 2B).^85^ Furthermore, recent studies on the time-dependent dynamics of mass transport into nanopipettes^86^ and characterization of nanopipette transport physics^87^ will benefit a wide range of nanoscale experimentation, including the synthesis of inorganic and organic materials^88^ and the stabilization of unusual polymorphs by confinement.^89^

Hybrid sensors can emerge from functionalized single-barrel or double-barrel nanopipettes. Edel and co-workers proposed a hybrid nanosensor for gated single-molecule transport by combining a nanopore-barrel and functionalized nanoelectrode-barrel acting as a field-effect transistor (Figure 2C).^90,91^ Such hybrid nanopipettes, with electrically and chemically tunable charge, can be employed for controlled molecular transport. Lastly, Ying, Long, Minteer, and co-workers demonstrated a wireless nanopipette-based electrode, supporting highly confined electric fields, that can favor bipolar redox reactions (Figure 2D),^92,93^ or sense protein–protein interactions.^94^

## High-Throughput Scanning Ion Conductance Microscopy

### From Nanopore to Nanoprobe

**Figure 3:**
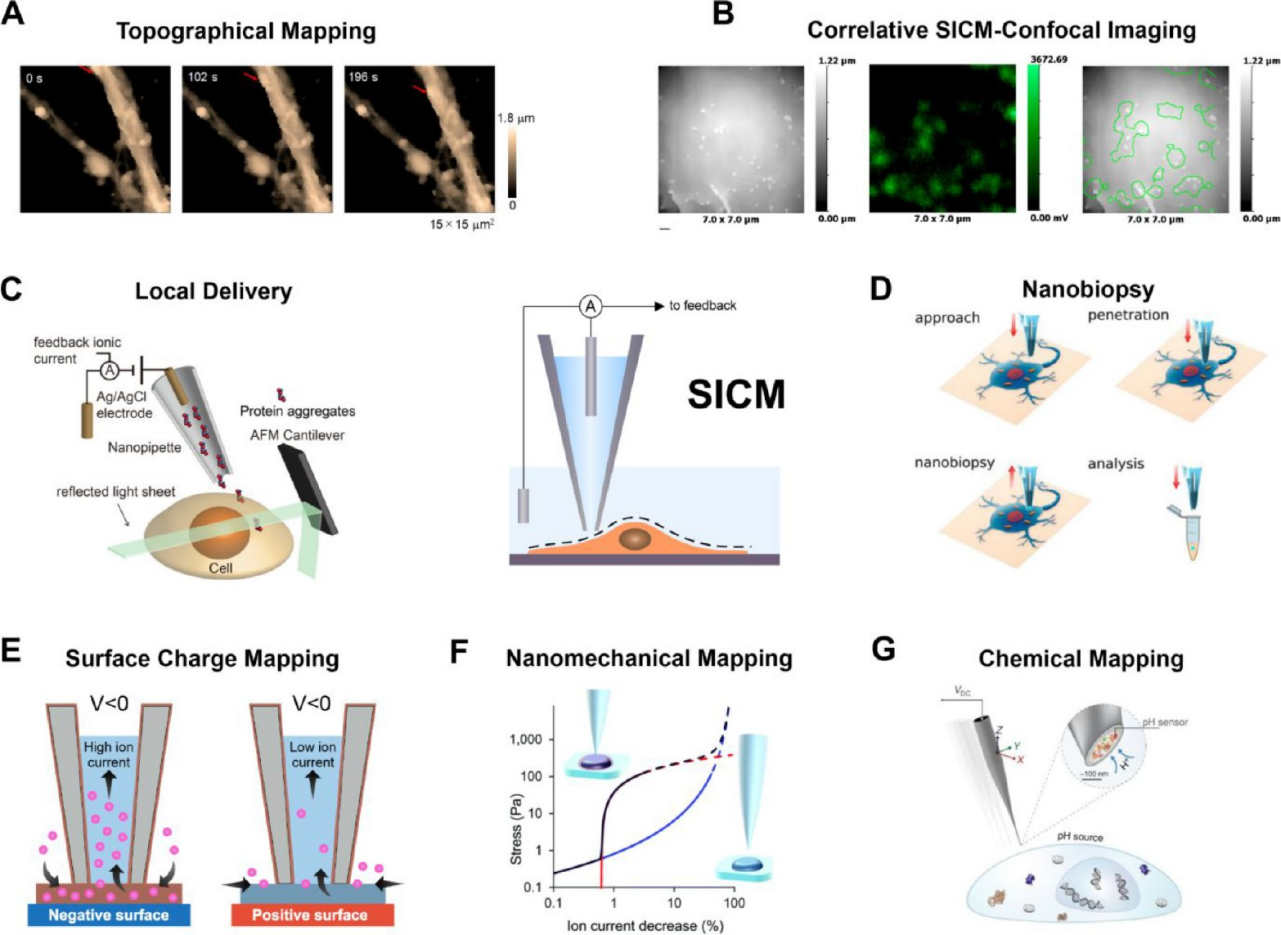
SICM depicted in the center for constant-distance imaging using the ion current as a feedback signal. The principal biology applications and mapping modes are shown in panels A–G. (A) Topographical mapping of single cells, reproduced from Takahashi, Y.; Zhou, Y.; Miyamoto, T.; Higashi, H.; Nakamichi, N.; Takeda, Y.; Kato, Y.; Korchev, Y.; Fukuma, T. *Anal. Chem.***2020**, *92* (2), 2159–2167 (ref (104)). Copyright 2020 American Chemical Society. (B) Correlative SICM-confocal imaging, reproduced with permission from Proceedings of the National Academy of Sciences USA Bednarska, J.; Pelchen-Matthews, A.; Novak, P.; Burden, J. J.; Summers, P. A.; Kuimova, M. K.; Korchev, Y.; Marsh, M.; Shevchuk, A. *Proc. Natl. Acad. Sci. U.S.A.***2020**, *117* (35), 21637–21646 (ref (105)) under CC-BY 4.0 license. (C) Local delivery of biomolecules combined with laser-sheet microscopy. Reproduced from Li, B.; Ponjavic, A.; Chen, W. H.; Hopkins, L.; Hughes, C.; Ye, Y.; Bryant, C.; Klenerman, D. *Anal. Chem.***2021**, *93* (8), 4092–4099 (ref (106)). Copyright 2021 American Chemical Society. (D) Single-cell nanobiopsy for cytoplasmic extraction. Reprinted from *J. Biol. Chem.***2018**, Vol. 293, Toth, E. N.; Lohith, A.; Mondal, M.; Guo, J.; Fukamizu, A.; Pourmand, N. Single-cell nanobiopsy reveals compartmentalization of mRNAs within neuronal cells, 4940–4951 (ref (107)) under CC-BY 4.0 license. (E) Surface charge mapping in aqueous solution and (F) nanomechanical mapping. Reproduced from Clarke, R. W.; Novak, P.; Zhukov, A.; Tyler, E. J.; Cano-Jaimez, M.; Drews, A.; Richards, O.; Volynski, K.; Bishop, C.; Klenerman, D. *Soft Matter***2016**, *12* (38), 7953–7958 (ref (108)) under CC-BY 3.0 license. (G) Extracellular pH mapping of single cells, reprinted by permission from Macmillan Publishers Ltd., Zhang, Y.; Takahashi, Y.; Hong, S. P.; Liu, F.; Bednarska, J.; Goff, P. S.; Novak, P.; Shevchuk, A.; Gopal, S.; Barozzi, I.; et al., *Nat. Commun.***2019**, *10* (1), 5610 (ref (109)) under CC-BY 4.0 license.

Scanning ion conductance microscope (SICM) is a scanning-probe technique where a glass nanopipette, filled with an electrolyte solution, is scanned over a sample immersed in an electrolyte while monitoring the ion current between a quasi-reference counter electrode (QRCE) inside the nanopipette and a QRCE in the reservoir.^95^ Ag/AgCl electrodes are typically used, which have high stability.^96^ For small bias and sufficiently high electrolyte concentrations, as the nanopipette approaches to approximately one radius away from a sample, the measured ion current decreases due to the restricted ion flow at the nanopipette opening (see below for situations where this is not the case). This signal dependence on the nanopipette–sample separation can be used as active feedback to map the topography of the sample. Korchev, Klenerman, and co-workers further refined the technique to allow topographical mapping of living cells immersed in culture media, broadening the applications of SICM to biology.^97^ SICM has been applied mainly in biological research,^98,30,97,99−102^ as depicted in Figure 3, but it is increasingly finding applications in electrochemical and materials research, which further expands its range of applications. A recent book^103^ provides a collection of historical developments and recent advances in SICM, as does an authoritative review.^18^

### High-Throughput SICM

In biological science, high-throughput methods are needed to dissect biological processes regulated by thousands of molecules whose concentrations quickly evolve as a response to specific stimuli or perturbations. Understanding the function and interaction of these biological entities at the nanoscale becomes critical to better address the bulk responses that we typically observe, although acquiring sufficient data to obtain the required statistical significance has been challenging.^110^ Recent advances in optical methods and SPM demonstrated the tracking of individual molecules in living systems and the mapping of single cells with high spatial resolution. These experiments generate a very large amount of data whose processing requires advanced and efficient data analysis pipelines.^111−114^ Compared to the subsecond image acquisition times of confocal laser scanning microscopy, SICM often requires several seconds or minutes to acquire a single topographical scan.^115^ More efficient hardware and software designs are required to develop high-speed imaging able to resolve fast dynamic processes. Furthermore, beyond imaging, the collection of sufficient samples/data to reach statistical significance still represents a significant challenge due to the lack of automation and the need for skilled operators. In the following sections, we will discuss the most recent developments to overcome these limitations and creative applications of SICM in single-entity research.

#### High-Throughput Imaging

High-throughput imaging enables measurement of the dynamics of cellular pathways, such as human genome positioning factors or DNA damage and repair in immune cells.^116,117^ In SPM, high-throughput atomic force microscopy (AFM) was achieved by developing smart image processing algorithms that allow multiparametric analysis of single entities and by developing high-speed imaging. Ridolfi et al. developed an AFM-based nanomechanical screening technique that allows nanomechanical and morphological characterization of thousands of individual extracellular vesicles.^113^ Konrad et al. developed a high-throughput pipeline based on AFM imaging with large fields of view and automated, multiparameter image processing for the investigation of nucleosome conformation.^112^ More generally, the development of high-speed AFM has allowed imaging of dynamic structures with unprecedented temporal, and spatial resolution and new processing algorithms are laying the groundwork to multiplexed and high-throughput analysis of biological dynamic processes.^118,119^

Conceptually similarly to high-speed AFM, high-throughput imaging in SICM is synonymous with high-speed scanning that can be achieved by optimizing the scanning routines and/or improving the hardware design. From the hardware perspective, the scanning speed is limited by the resonance frequency of the piezoelectric actuators and the time response of the electronics. Modern SICM is mainly based on the hopping mode, where the probe is retracted to a preset hopping height after each approach.^18,102,120−122^ As a result, the probe travels many micrometers along the *z* direction; thus, increasing the speed along *z* is the clear solution to improve imaging speed. Generally, piezoelectric actuators with resonance frequencies of tens of kHz and travel range of tens of micrometers are used for SICM. One limitation in using piezo actuators with higher frequency is imposed by the corresponding short travel range. The probe travel range needs to be at least 10–20 μm to scan convoluted biological surfaces (e.g., neurons), and this range limits the range of resonance frequencies available for the piezo actuator. Furthermore, the slow response of the active feedback results in an overshooting of the *z* piezo actuator, which becomes more prominent when the speed of actuation increases. Zhu et al. discussed recent hardware advances highlighting the benefits of using a second stacked piezoelectric actuator with a higher resonance frequency which compensates for the overshoot due to the slow feedback response.^18^ Another limitation to high-throughput SICM is the *x*–*y* scan area which needs to be small enough to reach high temporal and spatial resolutions.

From the software perspective, imaging speed has been improved by adopting optimized scanning regimes which drastically reduce the number of pixels acquired. A common example is the compression algorithm where the scan area is divided into small subarea regions of interest, and the resolution of each subarea is set according to the local roughness estimated by measuring the four corners of the subarea.^102^ This results in an increased temporal resolution due to flat subareas being imaged with a lower spatial resolution (higher speed) than rougher areas that contain the features of interest. Algorithms are being developed that allow the number of pixels acquired per experiment to be minimized (to decrease acquisition time) and reconstruct the final image in high resolution in a postprocessing step.^104,123,124^

Implementing an optical microscopy read-out is a further instrumentation solution to leverage the imaging throughput issue. Moreover, the optical readout (see dedicated section below) provides a complementary imaging of the objects (or function). Bednarksa et al. used correlative high-speed SICM and confocal microscopy to measure the kinetics of exocytosis of single granules of insulin from the top surface of single β.^125^ They synchronized the *z*-piezo actuator for nanopipette vertical positioning with another piezo used to vertically move the objective of a confocal microscope to achieve confocal autofocusing. The system allowed topography measurements of areas as large as 4 μm × 4 μm with a scanning speed as fast as 18 s/frame, while simultaneously acquiring confocal microscopy images of insulin granules inside and on the surface of single cells. The same group then imaged the kinetics of virus-like particle formation on the membrane of living cells, demonstrating that the particles can reach full size in 3–5 min.^105^

Simeonov and Schäffer^126^ used correlative ultrafast topographical mapping and multielectrode array recordings to simultaneously measure the morphology of cardiomyocytes and distribution of action potentials during a full contraction cycle. Navikas et al.^127^ integrated super-resolution optical fluctuations imaging to SICM to achieve correlative 3D microscopy of single cells. In their study, they used high-speed SICM with a pixel acquisition rate of 200 Hz to image cytoskeletal actinin dynamics on living cells. Hagemann et al.^128^ combined stimulated emission depletion and SICM to measure correlative surface topography and distribution of cytoskeletal actin. Gesper^129^ used SICM and fluorescence correlation spectroscopy to assess diffusion in cell membranes. Their findings revealed that cell surface roughness is unevenly distributed, with the plasma membrane above the nucleus being the smoothest.^130^

Leitao et al. developed a high-speed SICM based on high-bandwidth large-scale piezo actuator able to perform time-resolved, long-term topographical mapping of living eukaryotic cells.^131^ They demonstrated continuous surface topography measurement of large areas (80 μm × 80 μm) with good spatial resolution (512 × 512 pixels) and temporal resolution (from 0.5 s/frame to 20 min/frame depending on scan area). In earlier work, similar large scan areas were achieved by Zhuang et al. by developing a stitching algorithm able to “stitch” different scans in a postprocessing step.^123^

Recently, Zhuang’s group developed an SICM imaging mode using double-barrel nanopipettes and an adaptive sensitivity method to enhance the imaging rate.^132^ Wang et al.^133^ assessed the morphological and nanomechanical properties of intestinal tumor cells using high-speed SICM showing that highly metastatic cells exhibit unique topographical and nanomechanical features.

#### High-Throughput Single-Cell Manipulation and Measurements

The advent of next-generation sequencing considerably changed the meaning of high-throughput biology, massively scaling up the information generated with a typical assay. Scanning probe techniques are excellent tools for live investigations of cells, but they lack throughput due to a strong operator dependence and the intrinsic serial (nonparallel) acquisition system. Chen et al. recently published a ground-breaking study using FluidFM,^134^ an AFM with a microfluidic channel built in the cantilever, to longitudinally sample RNA from the cytoplasm of a living cell followed by transcriptomic analysis.^135^ With this approach, highly skilled operators perform hundreds of single-cell extractions in a semiautomated fashion, followed by molecular and data analyses. Similarly, SICM systems are still semiautomated and heavily rely on the operator skills, limiting upscaling possibilities.^97^

The past few years brought a series of advances that are increasingly moving SICM toward automation.^99^ Tóth et al. advanced the nanobiopsy technique, first introduced by Actis et al. to perform cytoplasmic extractions from single cells.^107,136^ An unsupervised algorithm based on self-organized maps (SOMs) allowed compartmental transcriptome profiling between different nanobiopsy locations within the same cell.^107^ Bury et al. used an adaptation of the technique to perform subcellular biopsies from human tissue with micrometer precision.^137^ Yu et al.^138^ used a similar approach to extract femtoliter volumes of cytoplasm from single cells for downstream time-of-flight secondary ion mass spectrometry (ToF-SIMS) analysis, expanding the applications of nanopipettes to *in situ* mass spectrometry.^139^ Similar techniques have been developed to deliver molecules inside single cells with high spatial resolution.^140−144^

Despite these studies being linked to high-throughput downstream biological analysis, the number of collectable samples is still limited by the lack of automation. Mukherjee et al.^145^ developed a fully automated nanofountain probe electroporation system based on angular approach SICM.^146^ A fully convolutional network was used to spatially localize cells in the optical field of view and to tag the position of nuclei. The system facilitated the successful delivery of a controlled amount of Cas9 nuclease to knockout specific genes in a variety of cell types in a fully automated manner. Automation partially overcomes some of the key problems with manual probe-based approaches, allowing the manipulation of tens of cells with the same nanopipette.^145^ Hu et al. used an Al_2_O_3_-coated nanopipette as an SICM probe to detect intracellular reactive oxygen species (ROS). Their system is based on a computer-vision algorithm for automatic detection of cell nuclei and lateral nanopipette positioning. The SICM-current feedback allows automated nanopipette vertical positioning on cell membrane while cell membrane penetration and intracellular ROS sensing is enabled by continuous monitoring of the ROS current.^147^

.A multiplexed localized electroporation device in conjunction with automated image segmentation and analysis based on artificial intelligence (AI) swiftly improved molecular delivery and transfection conditions for a variety of adherent and suspension cell types. The AI pipeline provides automated measurements of delivery/transfection efficiency and assists in the analysis of crucial cell morphological traits to pinpoint factors that promote high efficiency while also maintaining cell viability and health.^148^

These examples show how SICM could be coupled with AI algorithms to increase automation. Nonetheless, high-throughput and multiplexing can also be achieved, combining SICM with super resolution microscopy. Li et al. recently developed a novel multifunctional imaging system capable of high-speed 3D detection at single-molecule resolution within living cells and accurate delivery of single proteins to specific sites inside the cytoplasm or on the cell membrane.^106^ They combined a delivery system based on angular approach SICM with light-sheet microscopy to controllably deliver and track single molecules within single cells with unprecedented resolution and control. The technique was used to deliver amyloid-β aggregates on the surface of single macrophages to investigate the cellular response triggered by the aggregates. Similarly, Simonis et al.^140^ developed a portable and easy-to-build system to allow nanoinjections in single cells without the need for bulky and custom-made equipment, which would limit its use to equipped laboratories and highly skilled operators.

High-resolution multiparametric imaging enabled by SICM was achieved also for local pH mapping. Zhang et al. developed a label-free pH-sensitive nanoprobe made from a self-assembled nanomembrane that assembles in a zwitterion-like fashion at the tip of a nanopipette.^109^ The device was used for high spatiotemporal resolution monitoring of extracellular pH by precisely positioning the nanoprobe near the cell surface under SICM feedback control. These types of measurements used a double-barrel nanopipette, with one barrel for SICM and the other for potentiometric measurements.^149^ The same system was applied recently to quantify the chemical environment around single phytoplankton cells and to compare it to the conditions of bulk seawater, expanding the use of SICM to environmental sciences.^150^ All these studies underpin the potential of SICM as a noninvasive tool for single-cell manipulation and show the future directions that the technology will take to advance high-throughput biological investigations in life sciences.

### SICM as an Electrochemical Probe

The working principle of SICM for topographical mapping relies on the reduced ion flow (i.e., reduced ion current magnitude) generated by the proximity of a nanopipette to the investigated substrate immersed in an electrolyte solution. However, the nanopipette is also sensitive to other physicochemical properties of the substrate, such as surface charge^18,151,152^ or interfacial ionic concentration.^153^ When this is recognized and SICM is used as an electrochemical cell, for current–potential measurements, or potential pulse chronoamperometry, for example, it becomes a powerful multifunctional tool for surface and interfacial science, and electrochemistry in particular. With careful tuning and varying of the applied potential between the SICM tip and bath for each pixel in a scan, with the potential–time waveform aided by finite element method (FEM) modeling, it is possible to map the topography and other interfacial properties synchronously.^152^

When a glass nanopipette is immersed in an aqueous electrolyte solution, an EDL builds at the glass wall due to the negative charge generated by the deprotonation of silanol groups in solution, for a wide range of pH.^151,154^ As briefly highlighted above, the permselectivity of the conical nanopipette to cations (in this case) in solution generated by the EDL at the glass wall,^154^ leads to ICR, with strong rectification effects when the aperture diameter is in the same range of the Debye-length of the EDL.^154−157^ Similarly, charged surfaces immersed in an electrolyte solution possess an EDL, which affects ion transport through a nanopipette when positioned close to the surface, resulting in nonlinear current–potential responses,^151,158−160^ due to surface-induced rectification.^158,160,161^ A key aspect of these measurements is to be able to deconvolute surface charge and topography.^18,151^ A number of SICM modes are now available which recognize that the current is relatively insensitive to surface charge at very small bias and much more sensitive at extreme bias.^84,161^ Thus, quantitative SICM charge mapping makes use of multipotential measurements at each pixel.^84,161,162^

A recent technique, termed “quantitative surface charge conductivity microscopy” takes advantage of the potential dependence of surface-induced rectification. In this method, the same area is scanned under positive and negative bias (with the same amplitude) and the difference between the two maps is used to calculate surface charge density on the substrate solving the Poisson and Nernst–Planck equations which describe ion fluxes in the system.^163−165^ A double-barrel nanopipette was also used as the SICM probe to measure independently surface potential and topography, where one barrel was used to record the open-circuit potential while the other barrel was used to measure topography.^166,167^

Double-barrel nanopipettes have been used to probe ion transport at the nanoscale. Potentiometric SICM uses a multielectrode setup to probe conductance and transport across membranes.^18,168−172^ The technique allows conductance measurements at the nanometer scale across membranes with high spatial resolution. To improve the spatial resolution of SICM and to expand the range of applications to molecular/ionic flux sensing,^173−176^ ion channel probe-SICM has been developed, where a biological ion channel is inserted within a lipid bilayer formed at the nanopipette tip.

#### Applications in Material Sciences

**Figure 4:**
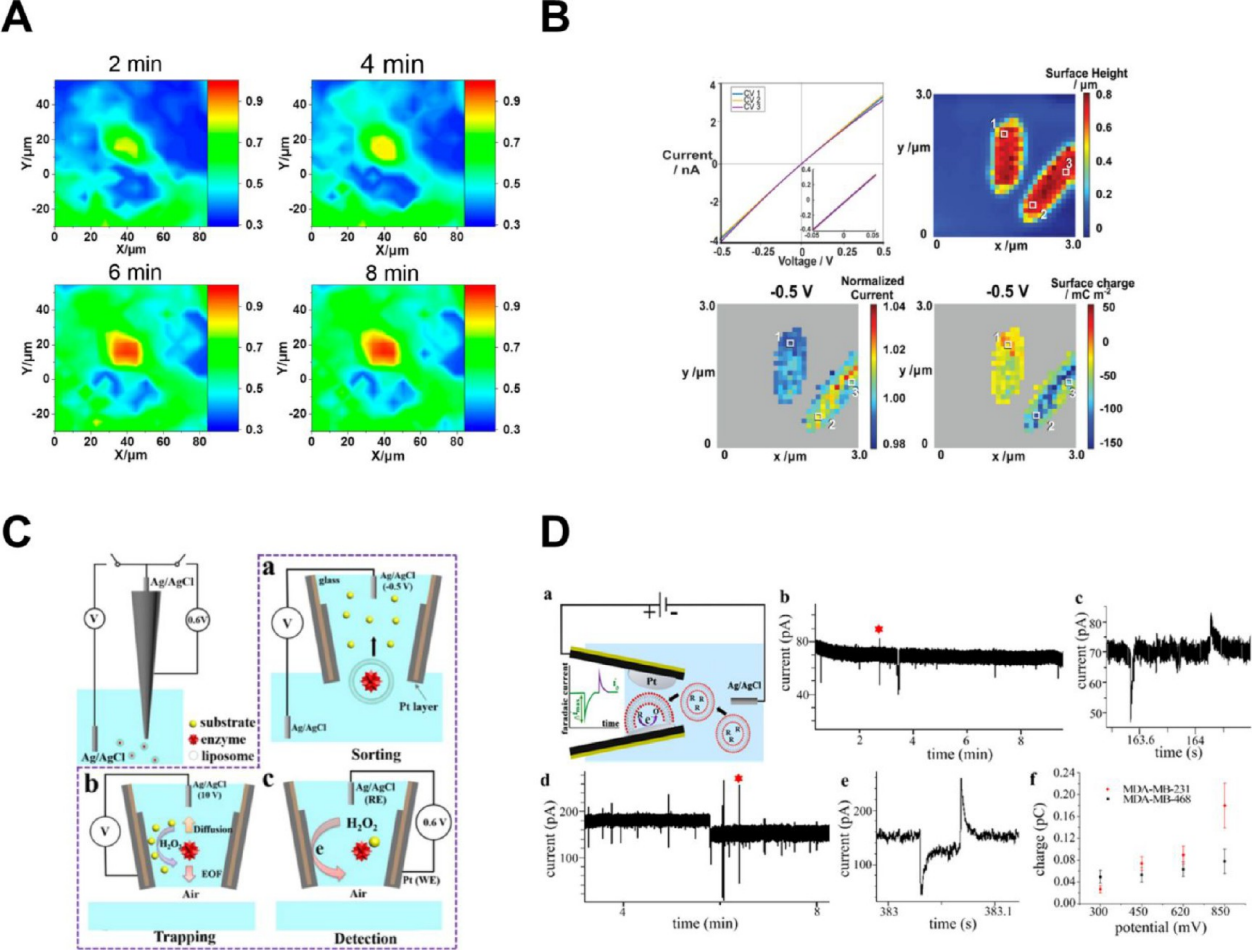
Applications of SICM- and nanopipette-based technologies for electrochemical sensing in material science and biology. (A) SICM images of Rhodamine B adsorbed TiO_2_ nanotubes after UV irradiation. Reproduced from Jin, R.; Ye, X.; Fan, J.; Jiang, D.; Chen, H. Y. *Anal. Chem.***2019**, *91* (4), 2605–2609 (ref (182)). Copyright 2019 American Chemical Society. (B) Surface charge mapping using SICM of *E. coli*. Reproduced from Cremin, K.; Jones, B. A.; Teahan, J.; Meloni, G. N.; Perry, D.; Zerfass, C.; Asally, M.; Soyer, O. S.; Unwin, P. R. *Anal. Chem.***2020**, *92* (24), 16024–16032 (ref (190)) under CC-BY 4.0 license. (C) Illustration of the electrochemical molecule trap based on a platinum-coated nanopipette and electroosmotic flow. Reproduced from Pan, R.; Wang, D.; Liu, K.; Chen, H. Y.; Jiang, D. *J. Am. Chem. Soc.***2022**, *144* (38), 17558–17566 (ref (194)). Copyright 2022 American Chemical Society. (D) Resistive pulse sensing based on nanopipettes. Reproduced from Jia, R.; Rotenberg, S. A.; Mirkin, M. V. *Anal. Chem.***2022**, *94* (37), 12614–12620 (ref (199)). Copyright 2022 American Chemical Society.

Measuring charge heterogeneities at solid–liquid interfaces is crucial to define local structure activity of materials.^18,151,152,177^ Surface charge measurements with SICM have been performed for various types of samples, from polymers to minerals, metal substrates, and electrodes.^18,152,178−180^ Zhu et al.^181^ mapped the surface charge of the clay mineral dickite, revealing a distinct pH-independent negative charge on the basal surface and a positive charge on step edges that increased as the bulk pH decreased from 6 to 3. Tao et al.^178^ measured localized surface charge of highly oriented pyrolytic graphite (HOPG) after the introduction of surface defects by plasma irradiation, finding that the topography of HOPG after irradiation showed more irregularities which were attributed to higher local surface charge compared to the pristine structure. Jin et al.^182^ found that locations with higher surface charge and stronger photocatalytic activity were responsible for irregular height profiles on TiO_2_ nanotubes. The photocatalytic degradation of organic pollutants (rhodamine B) by TiO_2_ has been investigated using SICM, providing an *in situ* technique for local investigation of photocatalytic processes (Figure 4A).

SICM can be also used for localized delivery of electroactive molecules to an ultramicroelectrode (UME).^179,183^ The effect of the substrate surface charge in regulating the delivery process has been investigated using a carbon fiber substrate.^180^ The role of electroosmotic flow induced by the local surface charge on the substrate in the delivery process was a key finding. This configuration has been used to create an artificial synapse, where the SICM probe delivers dopamine, locally and on demand, at >1000 different locations at a carbon fiber electrode. Heterogeneities in the response were linked to local properties, such as surface charge measured independently.^184^ Substrate permeability has also been investigated using SICM. Payne et al.^185^ demonstrated the possibility to measure permeability of porous membranes on a silicon wafer using SICM approach curves and FEM modeling. The dependence of the ion current on the local temperature around the nanopipette tip makes SICM suitable for localized thermometry, as recently reported for the temperature measurements on single nanoparticles, where temperature was measured with a sensitivity of 30 mK, and the method made use of nanopipettes as small as 6 nm in diameter.^186^

#### Applications in Life Sciences

The possibility to operate in physiological buffers makes SICM a powerful technique for measuring surface charge on single molecules and cells. Bioelectrical properties can affect biological processes including cell adhesion, antibody–antigen binding, cell–virus interactions, etc.^151^ The first application of charge mapping in biology used SICM to map surface charge at *Zea mays* root hairs, demonstrating their high negative surface charge density, and at human adipocytes, finding different surface charge distributions across their surface, including regions of positive charge that potentially pinpointed the location of key proteins (amino terminus) in the cell membrane, which are considered to mediate fatty acid uptake and perform other functions.^187^ SICM surface charge mapping has been extended to PC-12 cells,^162^ HeLa cells exposed to the thinning-agent dimethyl sulfoxide (DMSO),^188^ and human hair exposed to different conditioning treatments.^189^ Recently, Cremin et al.^190^ used SICM to map local ion properties of live bacterial strains (Figure 4B). Surface charge heterogeneities on the bacterial membrane were visualized for the first time, with significant differences in the ionic environments (surface charge and ion permeability) of Gram-positive and Gram-negative bacteria. This work was accompanied by detailed FEM modeling of the physicochemical properties of the bacterial cell wall, laying the groundwork for future understanding of the complex interplay between electrochemical microenvironment and physiological processes.

Single-cell electrochemical investigations using nanopipette technologies are not limited to the surface of the cell membrane but can be performed inside the cell with minimal disruption of cell viability.^143,191−193^ Pan et al.^194^ developed an electrochemical molecule trap based on a platinum-coated nanopipette where electroosmotic flow is used to accumulate molecules at the sensors and to impede the diffusion of molecules away from it. Their system allowed the measurement of the activity of single enzymes inside single cells (Figure 4C). Similarly, Liu et al.^195^ used a platinized carbon nanocavity inside a glass nanopipette to measure ROS in different regions of a living cell. This work expanded the range of intracellular electrochemical sensors based on nanopipettes for reactive oxygen and nitrogen species (ROS/RNS) developed previously and illustrated in Figure 4D for resistive pulse sensing.^196−201^ Similar electrochemical sensors based on carbon fibers have been developed to accurately measure the signal distortion due to the gap at the interface between the tissue and the sensor, providing benefit to research based on electrode–tissue interfaces.^202^

## High-Throughput Scanning Electrochemical Cell Microscopy

### Technical and Theoretical Developments

**Figure 5:**
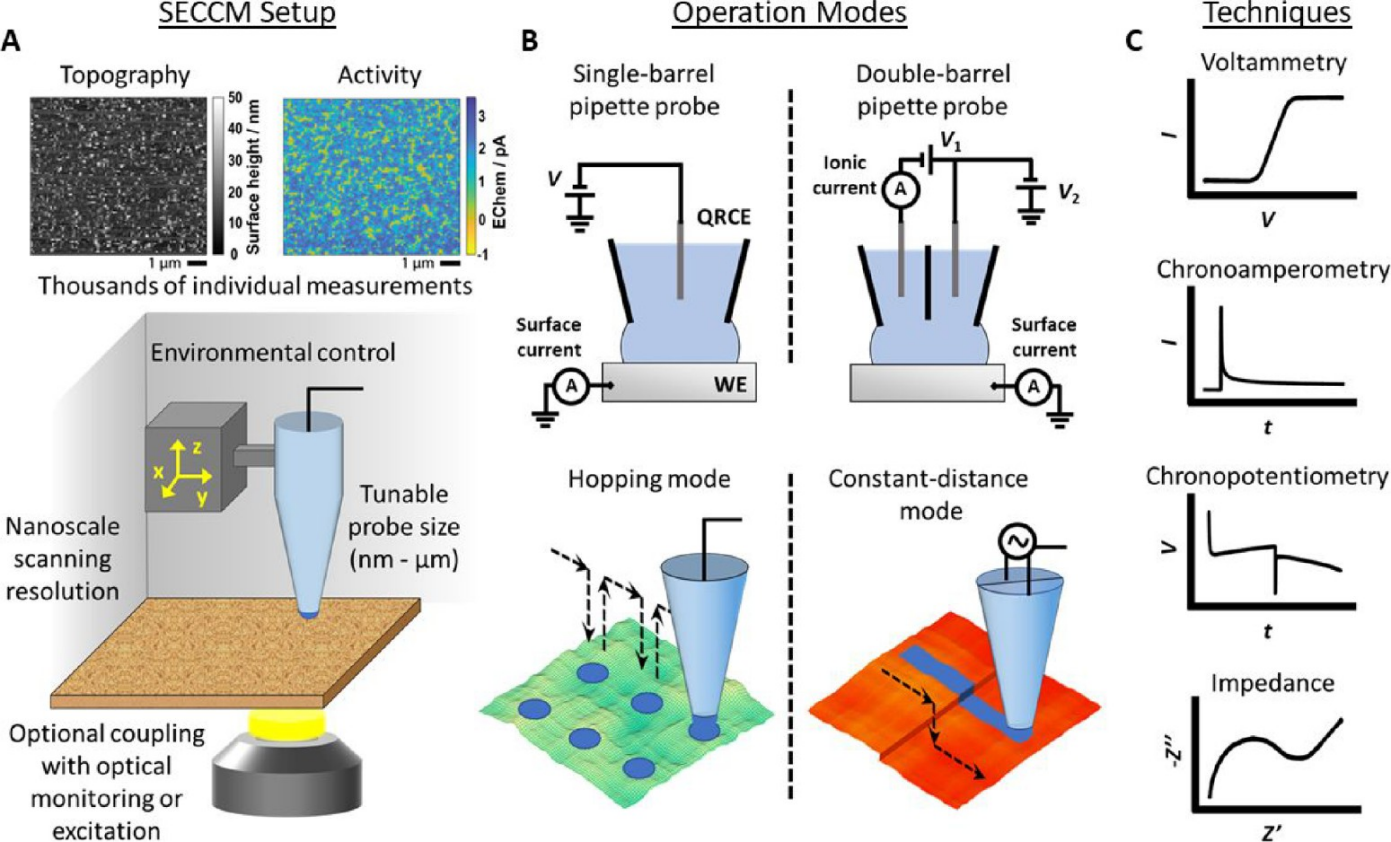
Schematic of the SECCM setup and imaging modes. (A) Experimental setup (bottom) and resulting measurement data (top). Simultaneous topography and activity mapping consisting of 7200 individual points. Reproduced from Wahab, O. J.; Kang, M.; Meloni, G. N.; Daviddi, E.; Unwin, P. R. *Anal. Chem.***2022**, *94* (11), 4729–4736 (ref (223)) under CC BY 4.0 license. (B) Operation modes showing the single- and double-barrel SECCM configuration (top) and the hopping and constant-distance modes (bottom). (C) Model measurement snippets, demonstrating various electrochemical techniques that can be applied in a localized manner using the SECCM platform.

SECCM is intrinsically a high-throughput analytical technique, able to directly interrogate nanoscopic areas of a target surface, accurately and at speed.^19,203^ Making use of the same pipet probes as SICM, the sample comes in contact only with the hanging electrolyte meniscus, formed at the pipet tip (Figure 5A). The wetted area of the substrate is typically of the same scale as the tip aperture and, as a result, it is both well-defined and easily tunable in size. The electrochemical activity is measured independently of, and synchronously with, the sample topography, allowing for unambiguous interpretation of recorded data. SECCM can perform a series of spatially separated localized experiments across a surface in an automated fashion.

Originally, double-barrel (or *theta* capillary) pipettes were used for SECCM experiments.^204^ In this configuration, a QRCE is inserted in each barrel, filled with the chosen electrolyte (Figure 5B). A voltage applied between the two QRCEs induces an ionic current flow between the barrels, through the hanging meniscus. The ionic current reflects any detectable change in the meniscus geometry. This is strongly revealed if the tip position is also modulated sinusoidally, normal to the surface, with the output of a lock-in amplifier, and the corresponding ion current is measured at the same frequency. Whether the tip position is modulated or simply approached, the ion current change upon meniscus contact can usually be detected with high sensitivity, thus permitting approach to any target surface, irrespective of its conductivity.^204,205^ Upon meniscus–surface contact, the extension of the vertical positioner reports on the sample topography. If the substrate is a conductor, it is usually connected as the working electrode (WE) and an additional voltage, applied between the QRCEs and the WE, facilitates the implementation of a variety of electrochemical techniques, within the confines of the miniature cell. Voltammetry experiments^206−217^ and chronoamperometry experiments^206,218^ are used to investigate the local electrochemical activity and to induce and monitor phase changes (Figure 5C). At the same time, the ionic current informs of any change occurring in the meniscus, e.g., the nucleation and growth of gas bubbles.^211^ With vertical tip position modulation SECCM, the surface topography can be tracked in a constant-distance mode (Figure 5B), where the probe does not break contact with the substrate.^204^ In this case, the potential versus the surface is kept stable, or swept while lateral movement is briefly interrupted.^205−207^

Recently, single-barrel pipet SECCM probes have been most popular.^19,219^ In this simpler configuration, originally called the scanning micropipette contact method,^220^ a single QRCE is loaded into the electrolyte-filled pipet (Figure 5B). It is kept at a certain potential versus the WE, so that a measurable current will flow upon meniscus–surface (WE) contact, at which point the vertical approach is halted. Single-barrel SECCM is used in a hopping mode protocol: the probe is approached to a predefined spot on the sample surface, a measurement is taken when in contact, and then the probe is retracted in order to be repositioned for the next spot.^221−224^ Potentiostatic techniques have been employed to explore heterogeneous electrochemical response at scales that reach tens of nanometers of spatial resolution. Resulting activity maps comprise thousands of individual points, each a voltammetric scan, for example, and reflect the technique’s high-throughput aspect.^223^ Additionally, potentiodynamic (galvanostatic) techniques have shed light into localized corrosion processes.^224,225^ Electrochemical impedance spectroscopy (EIS) measurements were also demonstrated, by combining SECCM with a frequency response analyzer.^221^

The easily customizable SECCM platform has allowed coupling to various instruments and methods (Figure 5A). Optically transparent substrates facilitated a user-guided, targeted scanning approach,^226−230^ or the interference-based optical monitoring of the electrode–electrolyte interface.^231^ Such substrates were used for the carrier generation-tip collection (CG-TC) SECCM method, mapping charge carrier diffusion over laser-excited areas.^228^ Other technical advancements have led to the capacity for regulating the electrolyte pressure inside the pipet probe^232^ or substituting with solid electrolyte.^233^ Environmental control during the experiment has been another major point of interest.^208,225,234−238^ Oil has been used to cover the substrate and restrict gas exchange from the meniscus,;^225^ while small containers connected to a gas supply were utilized to establish specific atmospheric conditions. Recently, SECCM measurements have been conducted inside a glovebox,^235,238,239^ and aided by a thorough understanding of the behavior of nonaqueous electrolyte media,^240^ they have set the groundwork for future work into battery materials.

Part of the popularity of SECCM as an analytical technique comes from a straightforward interpretation of the recorded data, based on the confinement of the reaction volume within a simple geometry. Theoretical and technical aspects of the experimental system have been covered since the introduction of the technique, with some further developments recently. The ohmic potential drop (*iR* drop) was studied in single- and double-barrel pipet configurations.^241^ Analytical treatments and simulations were considered. Another crucial element in the experiment is the stability of the Ag/AgCl QRCE, particularly during long measurements. Following previous scrutiny of typical SECCM configurations which provided recommendations for successive long-time imaging free from interference from the QRCE,^96^ the effect of Ag^+^ contamination on the estimation of open-circuit potential of aluminum alloy was investigated, and mitigation strategies for the use of a NaCl system were proposed.^242^ The stability of the pipet tip meniscus and its impact on surface imaging quality has been discussed elsewhere.^232^ The application of a controlled pressure to the back end of the pipet probe allowed the adjustment of the meniscus shape with respect to unique characteristics of the sample.

**Figure 6:**
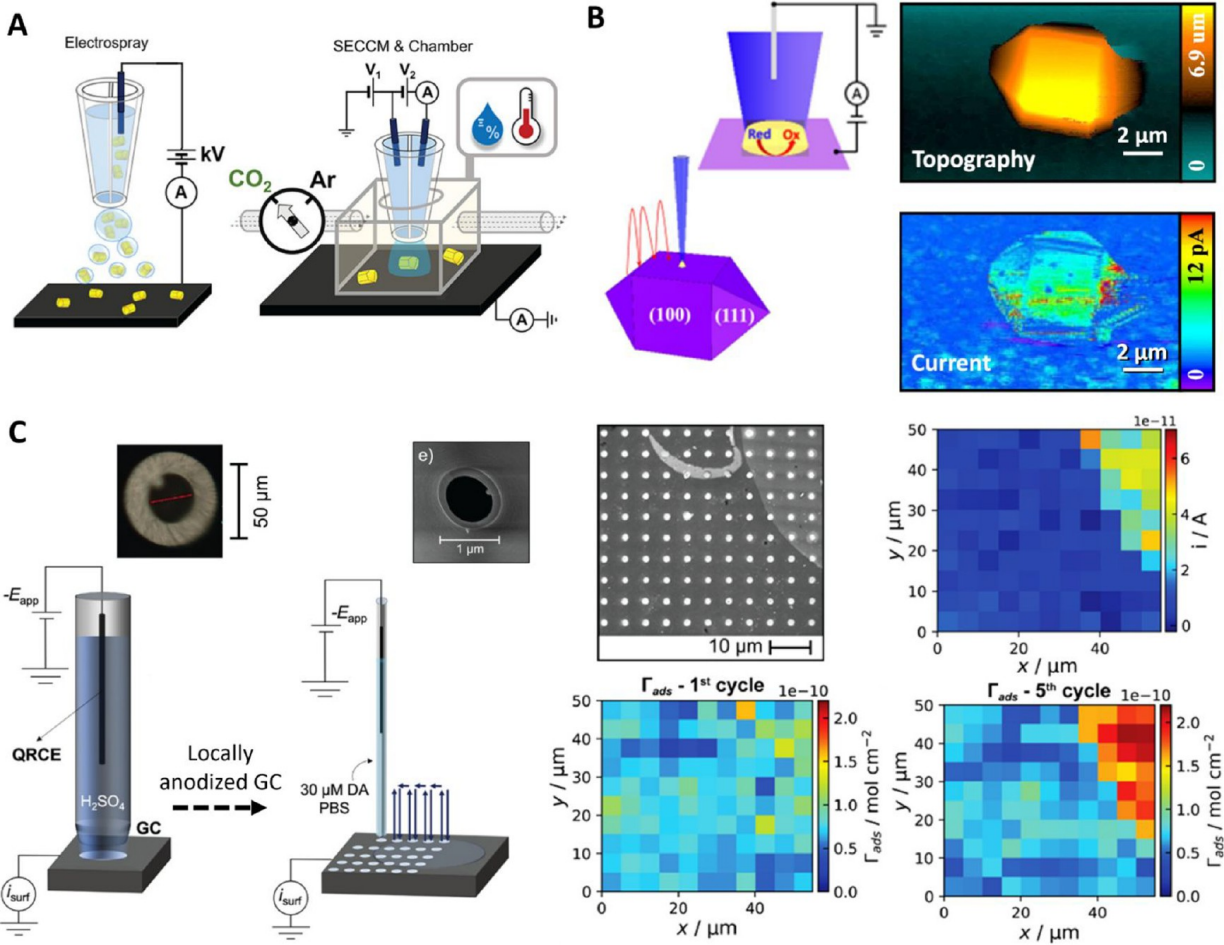
(A) Schematic of the pipet electrospray method for Au nanocrystal deposition; and the subsequent characterization of electrochemical CO_2_ reduction reaction, at individual nanocrystals, under a controlled environment. Reproduced from Jeong, S.; Choi, M. H.; Jagdale, G. S.; Zhong, Y.; Siepser, N. P.; Wang, Y.; Zhan, X.; Baker, L. A.; Ye, X. *J. Am. Chem. Soc*. **2022**, *144* (28), 12673–12680 (ref (208)). Copyright 2022 American Chemical Society. (B) Left: Illustration of SECCM mapping at a H-BDD single particle. Right: SECCM topography and current image, of a H-BDD single particle with the (100) face orientated upward, during Fe(CN)_6_^4–^ oxidation. Reproduced from Ando, T.; Asai, K.; Macpherson, J.; Einaga, Y.; Fukuma, T.; Takahashi, Y. *Anal. Chem.***2021**, *93* (14), 5831–5838 (ref (247)). Copyright 2021 American Chemical Society. (C) Left: Schematics of the SECCM device for local modification (anodization in H_2_SO_4_ solution) and to map the modified surface activity (with respect to DA electro-oxidation). Optical and SEM images of the respective pipet apertures are shown over the schematics. Right: SEM image of the SECCM scan area covering pristine (darker) and anodized (brighter) regions of the GC electrode; along with analysis of the spatially resolved voltammetric characteristics. Map of the current, *i*, obtained at the DA oxidation peak potential which shows that electroactivity is correlated to DA surface concentration (Γ_ads_) (first and fifth voltammetric cycle presented, respectively). Reproduced from Swinya, D. L.; Martin-Yerga, D.; Walker, M.; Unwin, P. R. *J. Phys. Chem. C***2022**, *126* (31), 13399–13408 (ref (249)) under CC BY 4.0 license.

A pipet-based electrospray deposition method was demonstrated with four different types of particles (gold octahedra, gold truncated ditetragonal prisms, copper nanorods, and polymer microspheres).^243^ The easily tunable experimental parameters led to deposition of single nanoparticles with a high degree of reproducibility and enabled their electrochemical characterization by SECCM in a follow-up study (Figure 6A).^208^ An equivalent circuit model for the SECCM electrochemical cell, under DC and AC conditions, was explored in another work,^244^ where an AC scanning regime resulted in enhanced surface topography mapping. AC perturbation to the experimental system was also utilized to conduct local EIS measurements, within the confinement of the meniscus cell.^221^

While a major focus of SECCM is on electron transfer processes, a new local electrochemical ion (proton) pump mode of SECCM has been developed to image proton transfer across membranes.^218^ In this mode, a double-barrel probe is used so that the meniscus cell can be landed on the membrane irrespective of its ion transfer characteristics. The driving force for ion transport is a potential applied between the SECCM probe and a counter electrode on the trans-membrane side. The technique was used to probe and visualize local proton transport across CVD graphene-on-Nafion membranes, for which proton transport activity was detected from atomic-scale defects, which were analyzed in detail to reveal the size from the current response, to larger cracks and tears. We anticipate that this technique could find myriad applications for imaging transport sites in membranes and coatings. Indeed, a recent application showcased the detection of nanometric pinhole defects across a multilayer aryl film on an electrode, formed by aryldiazonium reduction.^245^ As a model case for probing defects in protective coatings, detection sensitivity was examined with respect to the pipet and pinhole size.

### Electrochemical Measurements and Characterization

#### Popular Redox Reactions and Electrode Materials

Characterization of electrochemically heterogeneous surfaces, from the micro- to the nanoscale, is the primary focus of SECCM studies. The local degradation of an electrical double layer capacitor was investigated using HOPG as a substrate, and site-specific electrolyte decomposition was visualized via spatially resolved SECCM cyclic voltammetry measurements.^246^ SECCM was further employed to interrogate the electrochemical activity of Fe(CN)_6_^3–/4–^ at boron-doped diamond (BDD). The surfaces of single-crystal particles (Figure 6B) and polycrystalline electrodes were mapped,^247^ and the role of BDD surface terminations on the electron transfer rate was identified. Porous electrodes, made of compacted BDD particles, were also characterized,^248^ with respect to their surface activity, structure, and wetting.

Structure–activity relationships, governing the local electrochemical behavior of screen-printed carbon electrodes, were inspected with SECCM, followed by colocated correlative analysis via SEM and Raman spectroscopy.^216^ The electro-oxidation of dopamine (DA) was explored in detail by separately resolving the contribution of DA adsorption from that of intrinsic electrode kinetics. Further work on the DA system was carried out by a multiscale SECCM approach:^249^ glassy carbon electrode surfaces were functionalized utilizing a larger pipet probe and subsequently examined with a smaller one in imaging mode (Figure 6C). This study revealed how DA electrochemistry is affected by activated electrode surface sites. SECCM was also used by Schuhmann and co-workers to screen local activity of high-entropy alloys. By varying the pipet aperture size, the active site-specific oxygen evolution reaction (ORR) activities were visualized below micrometer scale via statistical analysis.^250^ The same group proposed a “ruler” technique, which determines the current profile of a single particle after exploiting focused ion beam marking and transmission electron microscopy (TEM) imaging to count the number of particles probed.^251^

Wang et al. developed a method to map the local potential of zero charge (PZC), through repeated SECCM approaches at a series of potentials,^252^ and noted correlation with local grain orientation. Further work by Wang et al. reintroduced the rarely used continuous scanning voltammetric mode of SECCM, originally developed by Güell et al.,^253^ and obtained similar results to Güell on (ferrocenylmethyl) trimethylammonium oxidation on the basal surface of HOPG.^207^ They further used the technique to reveal the grain orientation dependence of hydrogen evolution reaction (HER) on platinum, employing the powerful combination of SECCM and electron backscatter diffraction (EBSD), which is becoming an increasingly popular methodology.^254^

The local electrochemical current flowing through a thin organic coating is strongly dependent on the coating’s electrical conductivity. This was demonstrated from the nanoscale activity imaging of indium tin oxide (ITO) electrodes coated with blends of conductive (P3HT) and insulating (PMMA) polymer.^217^ Phase separation yielded conductive P3HT dots in a contiguous PMMA layer, presenting an ultramicroelectrode array for investigation by SECCM. The electron transfer kinetics for the oxidation of 1,1′-ferrocenedimethanol (FcDM) were determined by voltammetric imaging. Comparison to macroscale measurements revealed that the latter are dominated by bulk resistance effects.

Optimal sites for electrochemical hydrogen absorption on polycrystalline palladium electrodes were discovered using spatially resolved SECCM voltammetry in tandem with colocated EBSD analysis.^255^ High-index orientation grains demonstrated higher rates of electrochemical hydrogen absorption, and this methodology should be valuable in examining this process at a range of materials, for example in studies of the mechanical degradation of structural metals due to hydrogen embrittlement.

#### Corrosion

SECCM is becoming an increasingly popular technique in the field of metal corrosion. By confining the reaction within the probe meniscus, one can follow the dissolution at a nanoscopic scale and relate it to corresponding local variations in the material composition and structure. Importantly, corrosion-related processes can only be initiated upon, or after, meniscus contact, enabling the study of short time, transient, phenomena, and the initiation of processes. Surface electrochemical mapping, especially in conjunction with complementary colocated characterization, can provide a wealth of information about such structure–activity relations.

**Figure 7:**
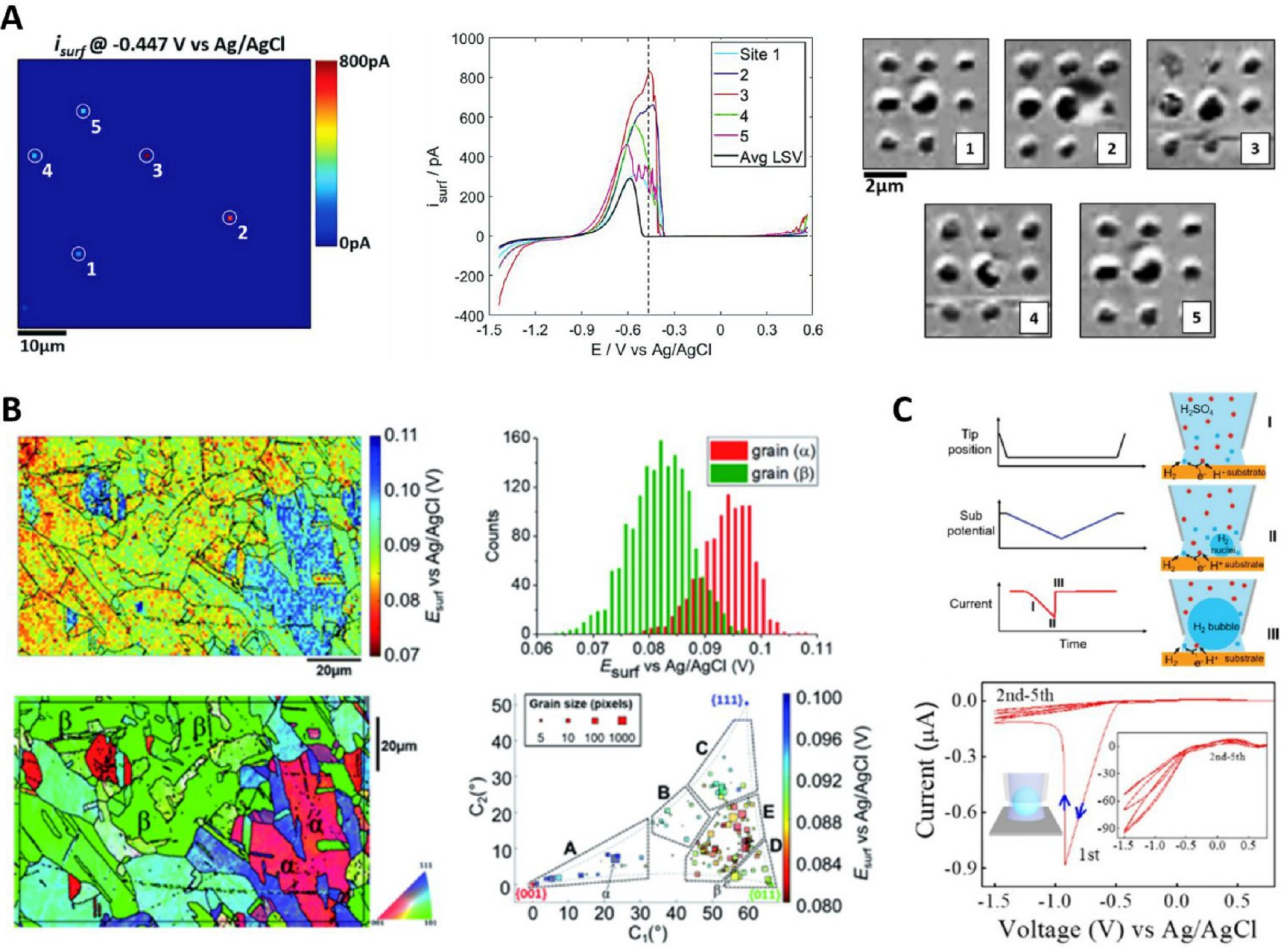
(A) Left and center: SECCM current map, at a WE potential of −0.447 V vs Ag/AgCl QRCE, obtained from 3600 localized voltammetry measurements of Fe dissolution; and *i*–*E* curves (scan rate = 2 V s^–1^) of the 5 highly active pixels labeled, versus the average response (black trace). The image in panel A is a snapshot at the potential indicated by the vertical dashed line. Right: electron microscopy images, after anodic dissolution of arrays of pixels, centered at the 5 active pixels. Reprinted from *Electrochim. Acta***2020**, Vol. 332, Yule, L. C.; Shkirskiy, V.; Aarons, J.; West, G.; Shollock, B. A.; Bentley, C. L.; Unwin, P. R. Nanoscale electrochemical visualization of grain-dependent anodic iron dissolution from low carbon steel, 135267 (ref (256)) under CC BY 4.0 license. (B) Left: snapshot of the required potential *E*_surf_ for the Cu dissolution process, measured during chronopotentiometry at 0.2s (top); with overlaid grain boundaries, taken from the colocated EBSD crystallographic orientation map (bottom). Right: statistical distribution of *E*_surf_ (top) extracted from grains α and β, of the EBSD map; and full grain orientation correlation analysis of *E*_surf_ at 0.2 s versus the average grain orientation. Reproduced from Daviddi, E.; Shkirskiy, V.; Kirkman, P. M.; Robin, M. P.; Bentley, C. L.; Unwin, P. R. *Chem. Sci*. **2020**, *12* (8), 3055–3069 (ref (225)) under CC BY 3.0 license. (C) Visualization of bubble nucleation with SECCM. Probing the electrochemical nucleation of single H_2_ bubble on a Pt surface via SECCM. Top: experimental parameters and voltammetric recording; and schematic of bubble nucleation and growth within the pipet tip, while in contact with the surface. Bottom: HER voltammetry with a 30 μm radius pipet, revealing blockage by H_2_ gas, after the first cycle. Reproduced from Liu, Y.; Jin, C.; Liu, Y.; Ruiz, K. H.; Ren, H.; Fan, Y.; White, H. S.; Chen, Q. *ACS Sens.***2021**, *6* (2), 355–363 (ref (267)). Copyright 2021 American Chemical Society.

A model exemplar system is the corrosion susceptibility of low-carbon steel in neutral medium.^213^ The anodic current response was probed and correlated with grain orientation via colocated EBSD. In addition, typically encountered MnS inclusions were identified in the sample, and their characteristic behavior was determined (Figure 7A). In subsequent studies, the dissolution of Fe in an acidic environment was targeted.^256^ The results were accompanied by density functional theory (DFT) calculations, and the corrosion susceptibility was thus rationalized by the energy required to remove one Fe atom at the surface of a lattice. A study of Zn corrosion^234^ was carried out, involving controlled atmospheric conditions (O_2_ or Ar atmosphere) with the SECCM meniscus surrounded by a dodecane oil layer deposited on the sample. The latter provided better control against excessive electrolyte spreading during cathodic processes, which can occur when there is a significant increase in local pH in the meniscus. Furthermore, the oil/aqueous electrolyte/metal system has been of special interest to the automotive industry and further diversifies the application of SECCM to interesting liquid/liquid/solid systems in general.

Corrosion of polycrystalline Al alloy AA7075-T73, under a NaCl medium, was examined in two related studies. First, the choice of the approach potential was scrutinized.^257^ For this system, the approach potential was shown to have an effect on subsequent electrochemical measurements. Experiments on the same system demonstrated dependence of corrosion behavior on the grain orientation.^258^ An extended pipet body was proposed to accommodate a three-electrode setup and reduce contamination from the Ag/AgCl reference electrode, which appears more problematic than typical in this particular system.

The crystallographic orientation-dependent corrosion of Cu has been studied, again utilizing dodecane oil, to achieve a triple-phase environment. Chronopotentiometry measurements were performed (Figure 7B) under O_2_ or Ar atmosphere to highlight key electrochemical processes underpinning localized Cu corrosion across a wide range of surface structural facets and grain boundaries identified by colocated EBSD.^225^ In a follow-up study employing voltammetric mode SECCM, the efficiency of an oil-soluble derivative of the classic Cu corrosion inhibitor benzotriazole was quantified for both anodic (copper dissolution) and cathodic processes, presented against structural variations of the surface.^259^ Another model system explored by a similar approach was the dissolution of Ag^260^ and its local reaction kinetics. Voltammetric mapping and additional colocated AFM characterization allowed extensive analysis of the dissolution mechanism and the prominent role of surface grain boundaries.

Extending the types of corrosion phenomena amenable to study by SECCM, the performance of the native oxide film over a bare metal base has been probed.^261^ The underlying Ni grain orientation matched to differences in the tendency of the NiO oxide film to break down at a particular Ni dissolution potential. As verified by colocated ToF-SIMS imaging, it was shown that locations with thinner oxide film were, counterintuitively, more resistant to breakdown. The results were explained and supported by a model that drew on classical nucleation theory.^262^

Combining both direct and alternating current polarization, single-crystal Mg corrosion was probed at the microscale.^263^ Electrochemical impedance spectra were acquired at confined areas across the Mg sample surface, and a distribution of relaxation time analysis revealed time-dependent interface processes. In a work that covers corrosion in archeological artifacts,^264^ purposefully prepared Ag–Cu alloy samples were studied in a variety of neutral solutions of different ion species. Localized electrochemical examination, across the alloy grain boundaries and general inhomogeneities, shed light onto the intergranular corrosion mechanism that would affect ancient artifacts of similar composition.

#### Phase Formation

SECCM has been widely used to study phase formation, owing to the ability to directly induce change within confined volumes, in a controlled manner. An attribute of SECCM is that measurements can be made at thousands of sites on a substrate, either under the same conditions or with conditions different at each spot or a portion of spots. This provides large data sets for detailed statistical analysis, and this aspect emphasizes the high-throughput nature of such SECCM measurements.

As a prototype of phase transition, gas bubble nucleation is commonly explored, both to progress theoretical understanding and improve real-life, critical applications. The objective could be to promote or restrict the evolution of gas, or to remove and guide it away from the active surface. The study of how bubble nucleation and growth is mediated by nanoscale dynamics is considered of major significance.^265^ Miniaturization of the working electrode surface enables the characterization of the nucleation of single nanobubbles.^266^ A classic experiment for the creation of a H_2_ nanobubble is to reduce H^+^, on a Pt disk nanoelectrode immersed in H_2_SO_4_ solution, by sweeping the electrode potential negative of E^0^ (H^+^/H_2_). With the increase of local H_2_ concentration, a bubble eventually nucleates and covers the electrode surface. This is mirrored in the recorded current, where an increase in current magnitude with driving potential eventually results in an abrupt decrease upon bubble nucleation.

A similar experiment has been applied in an SECCM configuration (Figure 7C),^267^ where confinement is naturally achieved within the meniscus cell. Similarities and differences—mainly related to the pipet aperture size and geometry—to the nanoelectrode setup were reported, and FEM simulations assisted in determining the relationship between the measured faradaic current and the local gas concentration. As mentioned above, an advantage of SECCM is the possibility of making measurements at several thousand different “electrodes”, defined by the meniscus contact, in one experimental run. Changing the electrode material from Au to MoS_2_, or structuring the surface,^268^ provided further insight into bubble nucleation and elimination dynamics and the overall bubble stability. The latter factor was assessed by chronoamperometric measurements at a fixed cathodic potential, resulting in periodic current oscillations, arising from the nucleation–growth–detachment lifecycle of a bubble. The effect of surfactants on electrochemically generated nanobubbles has been examined,^269^ and they were shown to promote HER and bubble formation on a Pt electrode, while also being critical in stabilizing H_2_ bubbles when using submicrometer radius SECCM pipettes. Electrochemically inert SiO_2_ nanospheres on a smooth glassy carbon surface were utilized as H_2_ bubble nucleants,^270^ when they were individually encapsulated and probed within separate SECCM landing sites. The peak voltammetric current was related to local gas supersaturation conditions over a range of nucleant sizes; and the role of the nanoconfinement geometry—between the nanospheres and the surface—was highlighted via theoretical analysis and FEM simulations. This study demonstrates the attraction of SECCM for nucleation studies, in being able to quantitatively assess a wide range of nucleation sites quickly in a combinatorial fashion.

NP nucleants dispersed on a catalytically inert substrate are representative of the typical nanocatalyst systems used in gas-evolving reactions. Individual Pt NPs on HOPG were encapsulated by a double-barrel pipet meniscus, and H^+^ reduction was performed.^211^ In this case, the pipet configuration allowed monitoring of bubble evolution via the ionic current flowing between the two barrels. Aided by FEM simulations, the relationship between the measured current peak and the local gas concentration was clarified. Probing distinctive particles in a spatially isolated manner is a recurring and powerful theme in SECCM studies.

Gas evolution was also explored with respect to the heterogeneous activity of the electrode surface. Nucleation and growth of H_2_ bubbles were monitored over the surface of polycrystalline Pt by a double-barrel probe in a native voltammetric mapping mode.^214^ Across the scanning pattern, the recorded response—related to the gas concentration and the activation energy required for nucleation—exhibited variations; however, it was notably not correlated with crystal grains or grain boundaries.

Moving to the solid phase, SECCM has been used to deposit preformed structures from solution or to form a solid phase from solution on a substrate. Electrochemistry can act as the driving force of phase change or simply assist in the process. SECCM serves as a powerful deposition tool in its own right as evidenced by its use to immobilize target catalysts onto special chip substrates for subsequent liquid-cell transmission electron microscopy.^271^ The nondestructive nature of the experimental procedure allowed the deposition of samples onto delicate substrates, by means of the residue left behind after probe retraction. The method played an important part in the successful *in situ* TEM observation of single catalyst particles during ethanol oxidation. In a similar fashion, tailored deposition of cobaloxime complex salts was achieved on carbon nanomembranes by means of SECCM arrays.^272^ The formation and the quality of alkanethiol self-assembled monolayers was observed electrochemically by SECCM.^273^ The reaction was confined within the area wetted by the nanopipette meniscus, while the process was monitored via the diminishing activity of the Fe(CN)_6_^3–/4–^ redox species.

SECCM has been particularly powerful in the study of the early stages of metal nucleation and growth. Building on initial work,^274,275^ Pt NPs were electrodeposited on carbon-coated TEM grid supports.^276^ A straightforward and high-throughput patterned deposition process provided separated ensembles of deposits, ready to be imaged under TEM and the structures related to experimental parameters (e.g., deposition potential, duration).

Cu electrodeposition is also a popular system for SECCM studies. Together with SEM, AFM, and XPS, SECCM was used to map the distribution of Cu nucleation activity on glassy carbon electrodes, to reveal a wide diversity of electrochemical activity which was subject to detailed statistical analysis.^277^ Cu nanostructures have also been produced by electrodeposition from SECCM nanopipettes.^278^ The limits of this electrochemical additive manufacturing (three-dimensional printing) process were explored, while it was fine-tuned to achieve feature sizes of just 25 nm.

In a case of indirectly driving phase change, CaCO_3_ crystals were precipitated from solution,^231^ within an SECCM meniscus, by electrochemically changing the local pH. Situated on a semitransparent ITO electrode, the landing spots were concurrently monitored with optical (interference reflection) microscopy, revealing spatially diverse growth regimes across the electrode surface. This work opens up new possibilities for studying the nucleation and growth of insulating materials (crystals, polymers) on surfaces using SECCM with direct optical visualization of the process. The optical microscopy set up could be further expanded to other optical techniques such as Raman microscopy.

#### Two-Dimensional Materials

Benefiting from unique structures and electronic properties, two-dimensional (2D) materials, including transition metal dichalcogenides (TMDCs, e.g., WS_2_, MoS_2_, and WSe_2_),^228,279−283^ graphene and graphene derivatives,^222,284,285^ hexagonal boron nitride (h-BN), etc.,^286^ have generated great interest in catalysis applications, especially in the area of photocatalysis and electrocatalysis. SECCM is ideally suited for studying the electrochemistry of such materials, as small flakes or regions of a 2D material can be targeted directly and the properties of those regions can be deduced by a range of colocated complementary techniques, e.g., Raman microscopy, AFM, etc.,^287^ to reveal the relationship between activity and electronic and structural properties of the material. Recent contributions showcasing electronic property manipulation—achieved by structural modifications, 2D support effects, and rational control of atomic defects—have provided insight toward understanding the interfacial charge transfer chemistry, ion transport, and carrier transport. These studies have tended to focus on simple outer sphere electron transfer processes, for which a range of one-electron transfer redox probes can be used. Ru(NH_3_)_6_^3+/2+^ is proving to be increasingly popular as a redox probe due to its stability and well-characterized electrochemistry and because the formal potential is close to the electroneutrality point of graphene and related materials,^288^ and so it is very sensitive to any changes in electronic structure of the materials studied. We cover such studies in this section, along with electrocatalytic processes at 2D materials. Photoelectrochemical studies are also of increasing importance in 2D materials,^289^ and some innovative measurements based on SECCM are reported in the next section.

SECCM and AFM were used to quantify the effect of layer number (∼5) on electron transfer kinetics in stacks of four different 2D TMDCs variants (MoS_2_, MoSe_2_, WS_2_, and WSe_2_).^290^ Faster kinetics on thinner stacked layers were observed in all cases attributed to a narrower electron tunneling barrier in low-layer stacked TMDCs, resulting from changes of band gap as a function of layer numbers of TMDCs. A similar experimental trend was obtained for chemical vapor deposition (CVD)-grown graphene on a copper substrate, where experiments on Ru(NH_3_)_6_^3+/2+^ were accompanied by a detailed, complementary computational and theoretical analysis of outer-sphere electron transter.^210^ Decreasing kinetics in the order of monolayer > bilayer > multilayer on copper was opposite to the trend for graphene on silicon oxide substrates,^253,287^ due to the key role of the copper substrate dominating the electronic properties. The relative rates on the monolayer versus bilayer graphene were shown to agree quantitatively with predictions for adiabatic electron transfer; the graphene layer acts as a barrier to ET at the electrode/electrolyte interface, with bilayer graphene having a bigger effect than monolayer graphene. These studies further highlight how the metal support has a profound effect on electron transfer at 2D materials,^291,292^ which was also demonstrated in SECCM studies of electrocatalysis of HER at h-BN comparing Cu and Au supports.^293^

One of the most exciting recent studies of outer-sphere electron transfer at graphene materials considered the electrochemistry of Ru(NH_3_)_6_^3+/2+^ at twisted bilayer graphene supported on h-BN, where the electron transfer kinetics were shown to be strongly dependent on the interlayer twist angle (θ_m_).^222^ θ_m_ was varied between 0.2° and 5.0°, and the electrochemistry was studied with SECCM. Around the “magic angle region”, ca. 1.1°, there is a significant increase in the density of electronic states (DOS) which leads to a significant enhancement in electron transfer kinetics. This study opens up a new dimension of topological defects in 2D heterostructures that can be explored with the combination of SECCM and correlative multimicroscopy.^294^

SECCM is a powerful method for detecting surface inhomogeneities and defects in 2D materials (e.g., cracks, grain boundaries, point defects, etc.). This was already highlighted for graphene on Nafion and films on electrodes in Technical and Theoretical Developments above. When electrochemical activity varies locally across a 2D material, this may be indicative of a key role of defect sites on the basal surface,^283,295^ and SECCM can be used to estimate the defect size. SECCM revealed that the basal planes of MoS_2_ (1H phase^296^ and 2H phase^283^) shows catalytic activity. The standard current density was measured to be about an order of magnitude lower on the basal surface than at the edge planes. SECCM was also utilized as an efficient patterning tool, to create localized controllable defects by local anodization of the surface (e.g., WS_2_ nanosheets and glassy carbon).^229,249^ These studies revealed the importance of defects in the structure as transport pathways, ranging from tears and cracks to atomic-scale vacancies.^245^

Heteroatom doping of 2D materials is an effective strategy for catalysis enhancement; charge accumulations—in pyridine, pyrrole, and quaternary N, across N-reduced graphene oxide (rGO)—are reported to increase catalytic activity.^284,285,297−299^ SECCM was applied to isolate the faradaic and nonfaradaic current, and the edge of N-rGO (rich in pyridine and pyrrole N) presented higher HER activity.^297^ Aided by DFT calculations, the pyridinic N in nitrogen and phosphorus codoped graphene was suggested to be the active site for HER.^285^ The combination of SECCM measurements and DFT calculations suggested that three-dimensional curved graphene possesses a high density of topographical defects and is able to accommodate the chemical dopants on the curved lattice, which exhibited higher electrocatalytic activity than surrounding flat regions.^284^

#### Photoelectrochemistry

SECCM is gaining in popularity for local photoelectrochemistry studies, as a tool to locally probe the activity of relevant photoelectrode materials. The charge injection yield at hematite films, during photoelectrochemical water splitting, was interrogated at the nanoscale with SECCM.^300^ Measurements were made before and after the addition of the electron hole scavenger, H_2_O_2_, at high spatial resolution along the electrode surface.

**Figure 8:**
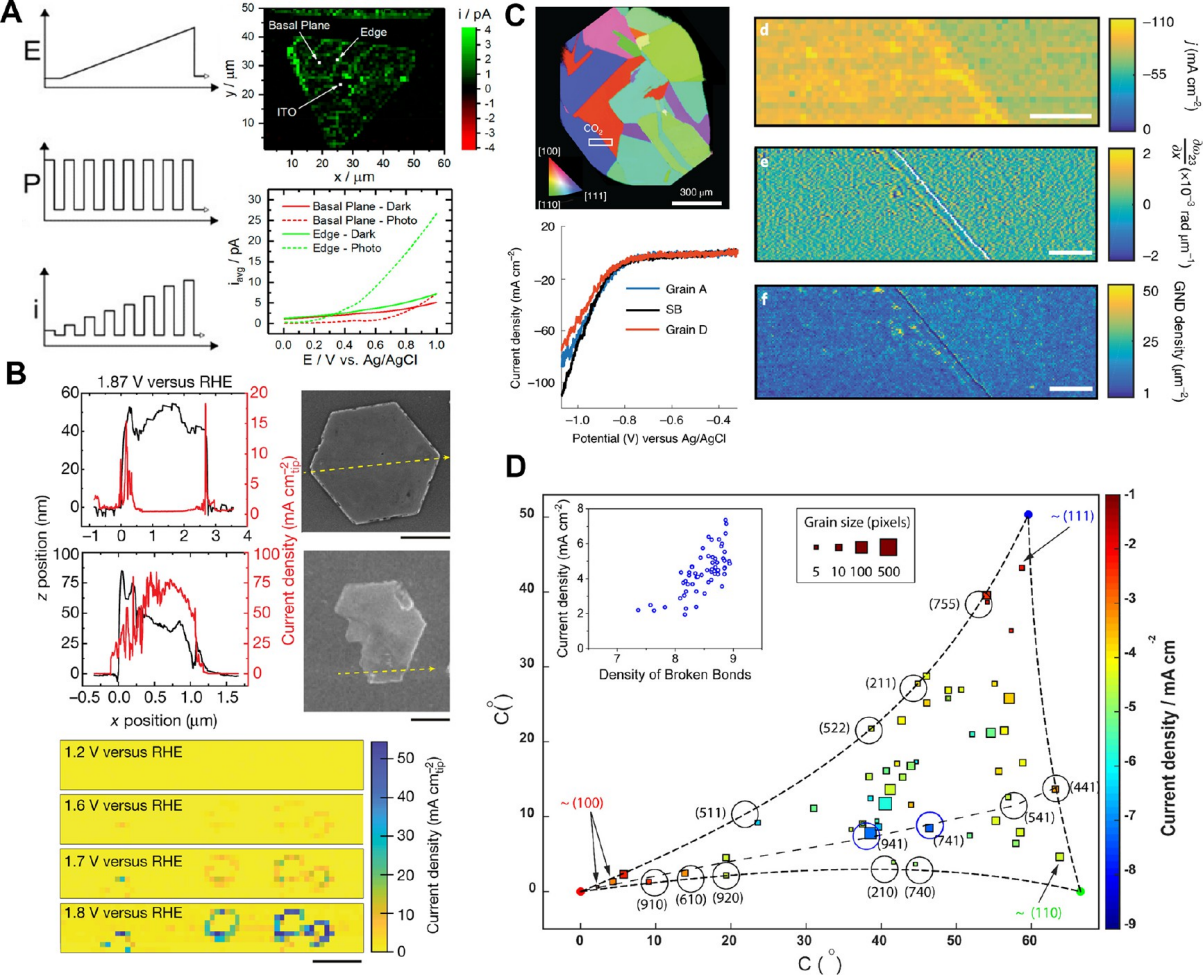
(A) Photoelectrochemistry. The SECCM applied potential, *E*, is swept, as the illumination, *P*, of the substrate is switched on and off, resulting in the measured local current, *i* (traces, left). Photocurrent image of individual MoS_2_ particle obtained via SECCM (top right), and averaged dark currents (solid lines) and photocurrents (dashed lines) observed at points across the basal plane (red) and edge plane (green). Reproduced from Strange, L. E.; Yadav, J.; Garg, S.; Shinde, P. S.; Hill, J. W.; Hill, C. M.; Kung, P.; Pan, S. *J. Phys. Chem. Lett.***2020**, *11* (9), 3488–3494 (ref (281)). Copyright 2020 American Chemical Society. (B) Electrocatalysis at layered Co(OH)_2_ plates. The oxygen evolution reaction (OER) current density as a function of position in scanning constant height mode for the particles shown on the right; the OER current density map obtained from hopping-mode SECCM movie at a series of voltage. Reprinted by permission from Macmillan Publishers Ltd.: Nature, Mefford, J. T.; Akbashev, A. R.; Kang, M.; Bentley, C. L.; Gent, W. E.; Deng, H. D.; Alsem, D. H.; Yu, Y. S.; Salmon, N. J.; Shapiro, D. A.; et al. *Nature***2021**, *593* (7857), 67–73 (ref (206)). Copyright 2021. (C) Top left: EBSD map of polycrystalline gold, with inverse pole figure legend of surface orientation. The white rectangle indicates the location of SECCM measurements in CO_2_ atmosphere. Bottom left: Representative linear sweep voltammogram extracted from grains and grain boundary. Right: Equipotential snapshot at −1.05 V vs Ag/AgCl obtained from SECCM movie, displaying spatially resolved eCO_2_RR activity. The lattice rotation gradient ∂ω_23_/∂x and derived geometrically necessary dislocation (GND) map of grain boundary. Reprinted by permission from Macmillan Publishers Ltd.: Nature Materials, Mariano, R. G.; Kang, M.; Wahab, O. J.; McPherson, I. J.; Rabinowitz, J. A.; Unwin, P. R.; Kanan, M. W., *Nat. Mater.***2021**, *20* (7), 1000–1006 (ref (237)). Copyright 2021. (D) Grain-resolved eCO_2_RR current density at −1.05 V vs Ag/AgCl plotted vs the corresponding average grain orientation relative to low-index orientation poles at polycrystalline Cu. Reproduced from Wahab, O. J.; Kang, M.; Daviddi, E.; Walker, M.; Unwin, P. R. *ACS Catal.***2022**, *12* (11), 6578–6588 (ref (236)) under CC BY 4.0 license.

Two-dimensional samples are of particular interest, and much work has focused on transition metal dichalcogenides sheets. An investigation into WSe_2_^230^ utilized an ITO substrate as a semitransparent contact and intermittent illumination from underneath in order to map the photoelectrochemical reaction rate with SECCM probes. Results showed a local activity dependence on the sheet thickness and the significance of defect features. A multiscale SECCM method allowed deliberate introduction of high-activity defects on WSe_2_ and subsequent photoelectrochemical characterization of the same sample.^229^ Along with topographical information and FEM simulations, the approach shed light on the morphology of the engineered defects and quantified local HER kinetics. Elsewhere, recording the spatially heterogeneous photoelectrochemical behavior of single or multiple layers of MoS_2_^281^ with SECCM complemented photoluminescence spectroelectrochemistry measurements (Figure 8A).

Coupling SECCM with a focused light source^228,279^ provided a methodology that was used to determine charge carrier diffusion within a WSe_2_ sheet. In this CG-TC SECCM configuration, the electrochemical pipet probe was scanned around an area of the substrate that was excited by illumination, collecting the photogenerated carriers. The spatial distribution of the carriers was revealed to be anisotropic, with faster in-plane than out-of-plane carrier transport, while hole confinement at structural defect sites was highlighted. Utilizing FEM simulations, the diffusion length could be estimated and related to the number of 2D sheets in the sample, with the bilayer WSe_2_ showing a very short diffusion length due to the high density of defects. The particular photoresponse attributed to heterojunctions of WSe_2_ and WS_2_ sheets was reported.^280^ Inspection of various arrangements aids understanding of the unique properties that arise in structures of that scale. Heterojunctions between 2D MoS_2_ and 3D Cu_2_O nanorods have also been probed.^301^

2D materials can serve as coatings for semiconductor surfaces, and other work^286^ examined p-Si/graphene and p-Si/h-BN interfaces. SECCM was used to determine the open-circuit voltage at the photoelectrodes, revealing the local electronic states of the material. The results of this study underlined the suitability of CVD-grown coatings for practical photoelectrode production. The performance of TiO_2_ nanotube structures was considered^302^ against proposed models for photoinduced charge separation within this architecture. Photocatalytic activity was recorded at different parts of a nanotube surface, with data supporting the orthogonal electron–hole separation model. A study was also conducted on the heterogeneous electron transfer performance of ITO electrode surfaces.^223^ This is commonly used as an electrode for photoelectrochemistry, including in some of the experimental setups reported in this section. Supported by FEM simulations, spatially resolved SECCM voltammetry of FcDM oxidation with a 50 nm diameter nanopipette showed heterogeneous patterns of electrochemical activity across the ITO surface, with only a tiny proportion of active (reversible) electron transfer sites, and a broad distribution of lower activity on the nanoscale. Interestingly, these nanoscale measurements suggested that macroscopic outer sphere voltammetry, on typical time scales, should be faster than observed in practice. This difference between nanoscale and macroscopic kinetics suggested that macroscale measurements are dominated by uncompensated resistances, most likely lateral conductivity in the ITO film under electrochemical operation, also observed in SECM measurements on a larger length scale.^303^

#### Electrocatalysis: Single Particles and Pseudo-Single-Crystal Screening of Structure–Activity

SECCM has been applied and proven to be a powerful technique to elucidate the structure–activity relationship of a wide range of electrochemically active materials in various electrochemical reactions. In Two-Dimensional Materials, we discussed some applications of SECCM on 2D materials, including electrocatalysis. Here, we describe how SECCM can be used to reveal the key role of particular local structures of electrocatalyst materials, such as the size,^304,305^ shape,^306^ porosity,^282^ facets,^208,212,236,307,308^ and morphology^309^ (e.g., particles vs particle ensembles). Surface features or defects (e.g., edge vs plane areas,^283,296^ grain boundaries,^237^ strains,^283^ vacancies,^283^ dislocations,^237^ etc.) can act as active sites and have significant contribution to the overall response. Identifying the local characteristics helps to understand the reaction mechanism and reveal the active sites for the rational design of highly efficient catalysts.

Conductive metal–organic frameworks (MOFs; e.g., Ni_3_(HITP)_2_,)^227^ are attracting considerable interest as electrocatalysts.^310^ The high spatial resolution of SECCM and intrinsically high mass transport for gaseous reactants^254^ was hugely beneficial in lifting mass transport limitations and revealing conductive MOFs orders of magnitude more active than previously recognized, paving the way to optimizing their use in larger-scale devices. SECCM, combined with other *operando* microscopies, was applied to investigate the oxygen evolution reaction (OER) activity of single-crystalline β-Co(OH)_2_ platelet particles.^206^ Simultaneous topography and OER current density maps were collected, demonstrating an inactive basal plane and dominant edge activity at pristine plates (Figure 8B). On the other hand, fragmented plates showed distinctive electrochemical activity due to gross defects.

SECCM has also been applied to electrodeposited amorphous films of molybdenum sulfides (α-MoS_*x*_), where spatially heterogeneous electrocatalytic activity was observed. This was attributed to variations in the nanoporosity of the thin film, after excluding contributions from composition, chemical structure, and surface roughness.^282^ This was revealed by analyzing the capacitive current in these measurements, to estimate the local electroactive (wetted) surface area *in situ* from the SECCM response.

SECCM offers a unique platform to isolate and analyze the electrochemical response of single metal NPs.^205^ In recent work, a template synthesis method, utilizing a nonconductive polycarbonate membrane with size-defined pore arrays, was used to create an array of electrodeposited Au tubules that acted as nucleation sites for the electrodeposition of isolated Pt particles, which in turn were characterized by SECCM.^209^ Patterns of Au nanocubes (NCs) and nano-octahedra (ODs), obtained via electrospraying,^243^ and expressing {100} and {111} crystal planes were screened for HER activity. The NCs exhibited higher electrocatalytic activity than ODs, in agreement with macroscale measurements. However, the NCs showed considerable particle-to-particle variations in catalytic activity, which is only revealed by targeted nanoscale measurements.^212^

In subsequent work, gold nanorhombic dodecahedra (RDs), ODs, and nanotruncated ditetragonal prisms (TDPs), expressing {110}, {111}, and {310} crystal planes, were investigated for the electrochemical CO_2_ reduction reaction (eCO_2_RR) by the same group.^208^ The RDs showed superior activity (expressed as turnover frequency, TOF) and selectivity compared with ODs and high-index TDPs. Results were in good agreement with the macroscale measurements, but the SECCM-derived TOF was an order of magnitude higher than the macroscale-derived TOF, due to the high mass transport rate of SECCM (see above).

SECCM was used to target borohydride oxidation at Au nanoparticles of controlled shape, which offered a variety of facets, and resulted in facet-dependent electrocatalytic activity.^306^ Other combinatorial analyses were performed in order to investigate the heterogeneity between, and within, single particles. Cu_2_O octahedrons with {111} facets showed higher activity than cubes with {100} facets. The poor inherent electric conductivity within Cu_2_O nanocrystals, resulting in low overall activity, was also underlined.^307^

Single hematite nanorods of different lengths, with tip and body part corresponding to {110} and {001} facets, respectively, were prepared to examine face-dependent OER activity.^311^ Increased OER activity was obtained at body sites, and longer nanorods with higher body share were beneficial in catalyst design. In a seminal work toward automation of SECCM operation, an optically targeted electrochemical cell microscopy method was established to perform guided measurements on individual Cu nanoparticles (dedicated regions of interest).^305^ In that case, no correlation was observed between electrocatalytic activity and particle size. In a study intending to probe the OER activity of single NiFe_2_O_4_ superparticles, a size-sensitive electrochemical response was reported.^304^ A particular composition effect (related to the Au content of NiFe_2_O_4_–Au particles) was only demonstrated at particle sizes smaller than 800 nm.

Experimental considerations were highlighted in studies of OER at single zeolitic imidazolate framework (ZIF-67)-derived particles.^215^ The Ag/AgCl QRCE was reported to be less stable at high current densities in alkaline solutions. A noninterfering Os^2+/3+^ complex was used as an internal reference redox couple (in solution) to correct for QRCE potential drift, and the voltammograms of encapsulated ZIF-67 particles were normalized with respect to the active surface area to exclude the current contribution from the substrate surface within the wetted area. A follow-up study explored the significant structural (e.g., morphology) and compositional (e.g., in spatial distributions of Co and O elements) changes of single Co_3_O_4_ particles at high current density in alkaline solution.^309^ Supported by TEM characterization, this study provided a view of structural transformations during electrochemical processes, at the single-particle level.

An interesting application of SECCM concerned the electrochemical analysis of Pt nanoparticles (70 nm in diameter) positioned at a buried interface between an HOPG support and a Nafion membrane. Electrocatalytic HER activity was investigated as a function of membrane thickness, with the highest activity at 200 nm thick films.^312^ This study opens up the use of SECCM to targeting the solid electrolytes, e.g., the electrode/electrolyte interface in a solid-state battery, or the electrode/electrocatalyst/membrane interface in a fuel cell.

SECCM is finding application in several studies of eCO_2_RR, with one example already highlighted above. The attributes of SECCM for such studies and the complementarity of the SECCM technique to related advanced voltammetric methods were recently reviewed.^313^ SECCM provided evidence to support the direct link between local configurations of Sn/rGO and eCO_2_RR performance.^314^ Activity at three sites was considered: the Sn particles, the rGO surface, and the boundary between Sn and rGO. A controlled atmosphere of Ar or CO_2_ during the experiment was essential in order to confirm that the interface between Sn particles and rGO promotes CO_2_ reduction and suppresses proton reduction to hydrogen. Additionally, a synergetic effect in the catalytic mechanism of Sn/rGO was emphasized, with CO_2_ activity measured from two sources: (i) directly, from the CO_2_ adsorbed on the Sn surface, and (ii) indirectly, from the CO_2_ adsorbed on oxidized function groups of rGO, continuously migrating to the Sn surface to supply the reaction and enhance the catalytic activity.

SECCM with colocated EBSD maps has been used extensively to screen the local response over multiple crystallographic orientations and grain boundaries of pseudo-single-crystal materials (e.g., polycrystalline metals that can be characterized by EBSD) for a range of processes (e.g., HER, eCO_2_RR, OER, etc.). The combined, high-throughput approach has proven to be a powerful tool toward interpreting structure–activity relations. Mariano et al. studied eCO_2_RR on polycrystalline gold^315^ and reported enhanced eCO_2_RR activity around grain boundaries of specific geometries, which can be attributed to an altered lattice strain and increase of dislocation density. In a follow-up work, SECCM and high angular resolution EBSD were employed to investigate the origin of enhanced electroreduction activity at the slip band around the Σ3 grain boundary region and defect-rich areas (Figure 8C).^237^ The results call attention to dislocation migration and slip bands fulfilled with defects—other than lattice strain—that give rise to locally enhanced activity. More recently, correlations between the local crystallographic orientation of polycrystalline copper and eCO_2_RR activity were investigated, with a view to create a library of facet indices (Figure 8D).^236^ Results showed an increasing eCO_2_RR activity in the order of (111) < (100) < (110). The facets (941) and (741), in particular, displaying higher step and kink feature density, resulted in higher electroreduction activity. A naturally formed copper-passivating layer (Cu(OH)_2_, CuO, and Cu_2_O) shows strong grain-dependent stripping behavior. Sequential cyclic voltammetry cycles verified that the surface oxides could be removed down to an electrochemically undetectable level after a single cycle pretreatment, which eliminates contribution from the passive layer in studies of eCO_2_RR at copper.

Identifying intermediates in electrocatalytic processes is an important goal. Quad-barrel SECCM tips are available, which contain up to 2 solid electrodes that can be used to detect the products (and, in principle, intermediates) of local surface processes within the SECCM meniscus.^316^ Another interesting development is the use of fluorophores within the tip to identify reactive oxygen species, produced in the tip, during the oxygen reduction reaction.^317^ This is a platform that could readily be expanded to other species and methods of detection.

#### Battery Electrode Materials

**Figure 9:**
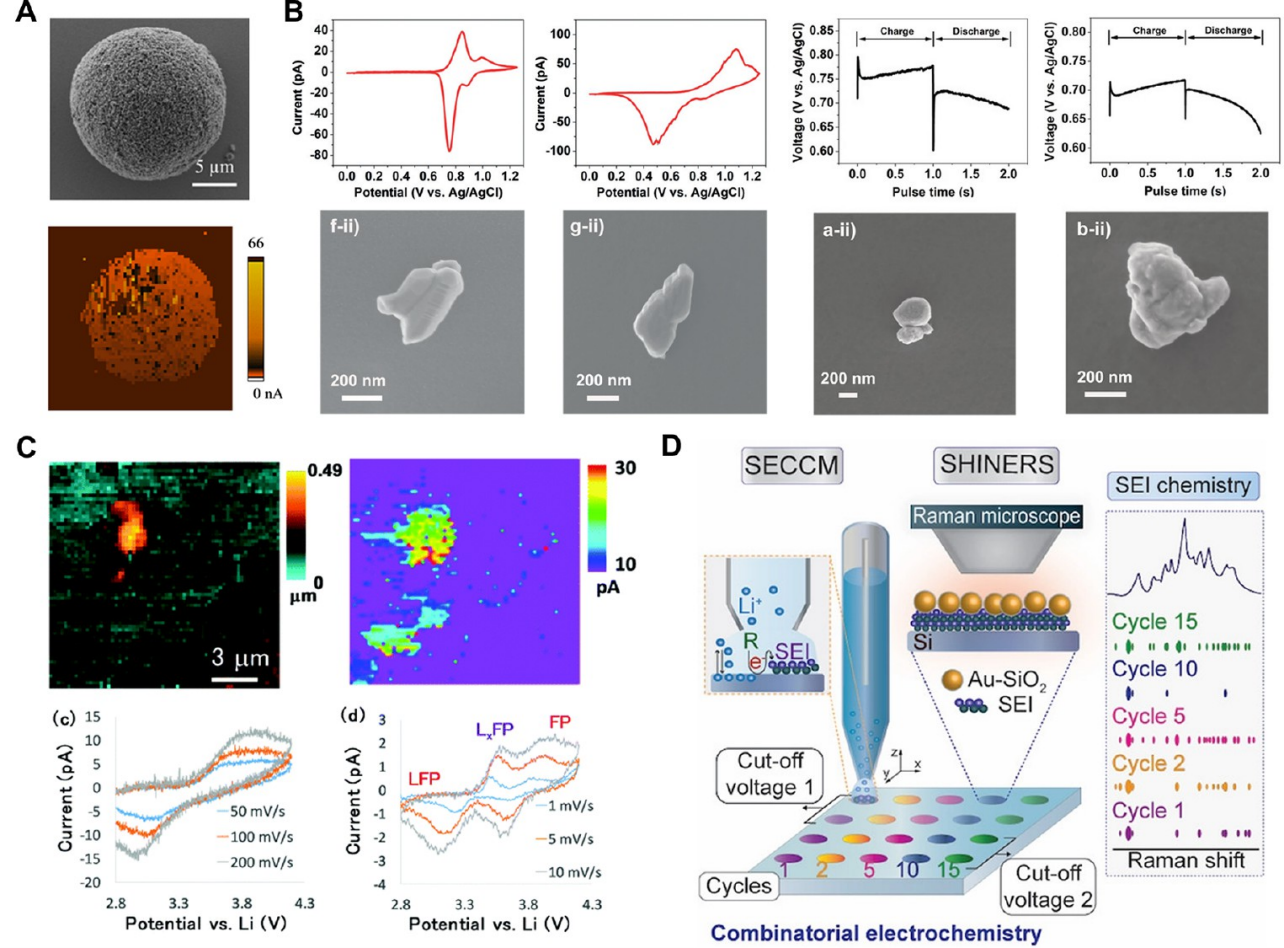
(A) SEM image and SECCM charging current response of an individual LiFePO_4_ particle. Reproduced with permission from Scanning electrochemical cell microscopy for visualization and local electrochemical activities of lithium-ion (de) intercalation process in lithium-ion batteries electrodes, Kumatani, A.; Takahashi, Y.; Miura, C.; Ida, H.; Inomata, H.; Shiku, H.; Munakata, H.; Kanamura, K.; Matsue, T. *Surf. Interface Anal.*, Vol. 51, Issue 1 (ref (320)). Copyright 2018 Wiley. (B) Cyclic voltammograms and galvanostatic charge/discharge curves of individual LiMn_2_O_4_ particles along with corresponding SEM images revealing their varied morphology. Reproduced from Correlative Electrochemical Microscopy of Li-Ion (De)intercalation at a Series of Individual LiMn_2_O_4_ Particles, Tao, B.; Yule, L. C.; Daviddi, E.; Bentley, C. L.; Unwin, P. R. *Angew. Chem. Int. Ed.***2019**, Vol. 58, Issue 14 (ref (321)) under CC BY 4.0 license. (C) SECCM topography and current response of Li^+^ (de)intercalation at a LiFePO_4_ particle. Cyclic voltammetry at the LiFePO_4_ particle, using different scan rates and showing stages of the Li^+^ (de)intercalation. Reproduced with permission from Takahashi, Y.; Yamashita, T.; Takamatsu, D.; Kumatani, A.; Fukuma, T. *Chem. Commun. (Camb.)***2020**, *56* (65), 9324–9327 (ref (238)), with permission of the Royal Society of Chemistry. (D) Schematic of automated SECCM for combinatorial electrochemical screening of the SEI formation, with correlative chemical analysis through SHINERs and Raman microscopy. Reproduced from Dynamics of Solid-Electrolyte Interphase Formation on Silicon Electrodes Revealed by Combinatorial Electrochemical Screening, Martin-Yerga, D.; Milan, D. C.; Xu, X.; Fernandez-Vidal, J.; Whalley, L.; Cowan, A. J.; Hardwick, L. J.; Unwin, P. R. *Angew. Chem. Int. Ed.***2022**, Vol. 61, Issue 34 (ref (239)) under CC BY 4.0 license.

SECCM provides high spatial resolution, *in situ* imaging to break down the averaged response at a macroscopic battery electrode, from a combination of active materials, binder, and additives, enabling investigation of battery materials down to the single-particle (entity) level.^318^ The performance of lithium-ion battery (LIB) cathode materials has been the subject of a number of studies. SECCM was first employed (in aqueous solution) to investigate localized redox activity on composite LiFePO_4_ (LFP) in 2014,^219^ revealing heterogeneous reaction rates depending on the local composition. More recently, SECCM was used to evaluate metal oxide coating effects on Li^+^ (de)intercalation at LiCoO_2_.^319^ It was found that ZrO_2_ coating can improve cycle durability at the expense of reaction rate. Ion transport at isolated LiFePO_4_ particles was also investigated, demonstrating fairly homogeneous Li^+^ deintercalation on a single particle (Figure 9A).^320^ Meanwhile, Tao et al. employed SECCM to investigate a series of LiMn_2_O_4_ particles by cyclic voltammetry and galvanostatic charge/discharge methods (Figure 9B).^321^ The particles showed different behaviors with respect to particle shapes and structures, with results that are relevant to battery material design.

Bentley et al. explored the application of organic carbonates (propylene carbonate, dimethyl carbonate, and ethylene carbonate), as a model class of aprotic solvents, for SECCM measurements under conditions comparable to those of battery operation.^240^ Assessing the I^–^/I^3–^ and I^3–^/I_2_ couples and fabricating polypyrrole arrays confirmed the stability and consistency of these solvent systems in SECCM configuration, in turn providing confidence for future SECCM work in battery applications. Takahashi et al. first performed SECCM experiments inside a glovebox and used typical commercial LIB electrolytes (1 M LiClO_4_ in ethylene carbonate/diethyl carbonate, vol. 1:1) to investigate the facet-dependent Li^+^ electrochemical reactivity and diffusion coefficient at Li_4_Ti_5_O_12_ thin-film electrodes.^238^ They also achieved fast charging/discharging of single LiFePO_4_ particles at a scan rate of 1 V s^–1^ and characterized a metastable state of Li_*x*_FePO_4_ (Figure 9C).

Three different types of HOPG, having different step densities and step heights, were utilized as a model system to investigate solid electrolyte interphase (SEI) formation with SECCM in a glovebox. SEI is formed at the surface of LIB negative electrodes due to reduction and decomposition of electrolytes, and this dynamic process is crucial for battery performance. Slow scan rate SECCM voltammetry revealed that step edges promote electrolyte reduction resulting in a more passivating SEI layer than on the basal plane.^235^ In recent work, SECCM measurements were coupled with enhanced Raman spectroscopy monitoring via shell-isolated nanoparticles (SHINERS technique, Figure 9D).^239^ Two different aprotic electrolyte systems (ethylene carbonate/ethyl methyl carbonate, vol. 1:1; and propylene carbonate) were used to perform local cyclic voltammetry on (111)-facet silicon. SECCM/SHINERS coupling sheds light onto the stages of a continuously evolving SEI through tweaking the number of cycles and potential cycling range. Finally, the charge transfer resistance on individual LFP particles was quantified, as a model system for exploring battery charging/discharging at high rate.^322^ The results affirmed the significance of discharge level and electrolyte solution type (aqueous vs organic solvent here).

## Optical Microscopies in Electrochemistry

Optical microscopy methods have long been used in physics and biology, for example, to quantify the action of forces by monitoring the motion of objects and to identify the biological functions or structure of subentities of living microorganisms. Optical microscopies have been considered less in chemistry or electrochemistry. One of the reasons is that the visible region of light used to obtain an optical image is not usually the most informative spectral range for identifying chemical structures or chemical transformations. This view has changed considerably over the last decades due to progress in (a) the synthetic design of molecules or nanoobjects with switchable optical properties in the visible range; (b) the development of highly sensitive and rapid photodetectors, e.g., able to detect the ultimate limit of a single photon; and (c) the development of (instrumental or computational) strategies enabling imaging at higher resolution (such as super-resolution microscopy) and higher sensitivity (even label-free).

### Overview of Operational Principles

#### Operational Principles

For details of the progress and the operational principles and applications of optical microscopy for imaging electrochemical systems, we refer to a number of recent reviews.^23,24,323,324^ Here we focus on new methodologies able to unravel greater levels of complexity in terms of electrochemical processes/information, including visualizing simultaneous phenomena, as well as new experimental and image-processing methodologies.

**Figure 10:**
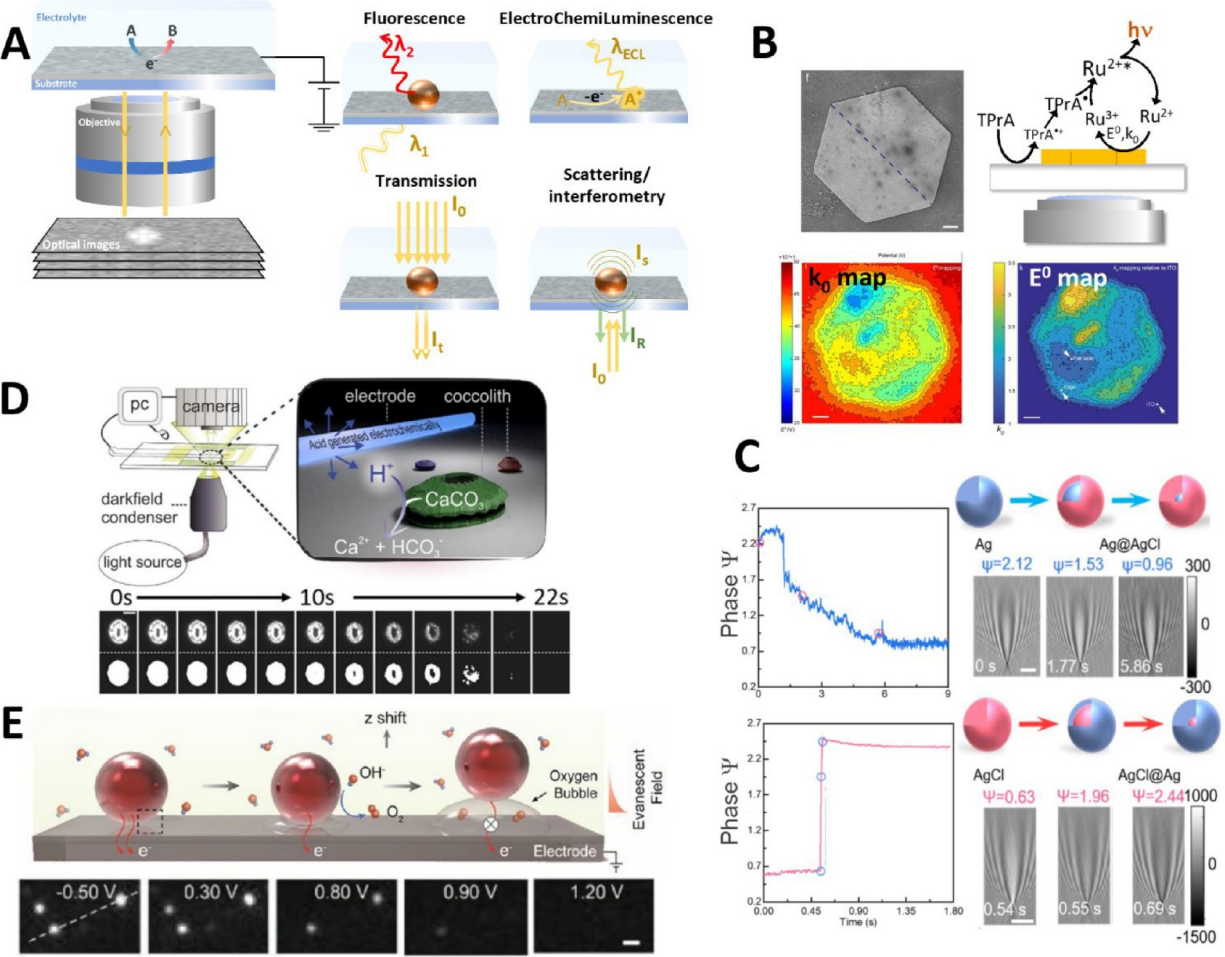
(A) General principles of optical microscopy imaging of electrochemical processes, with schematic setup and some selected types of detection based on the phenomenon at the origin of the imaging process. (B) Single-photon ECL imaging of Ru(bpy)_3_^2+^ oxidation at an Au hexagonal nanoplate: SEM micrograph with corresponding kinetic and thermodynamic mappings of Ru(bpy)_3_^2+^ oxidation. Reproduced from Operando Imaging of Chemical Activity on Gold Plates with Single-Molecule Electrochemiluminescence Microscopy, Dong, J.; Xu, Y.; Zhang, Z.; Feng, J. *Angew. Chem. Int. Ed.*, Vol. 61, Issue 14 (ref (347)). Copyright 2022 Wiley. (C) Implementing optical phase imaging in scattering interferometric microscopy to track the electrochemical conversion of Ag into AgCl NPs and vice versa. Reproduced from Wu, G.; Zhou, X.; Lv, W. L.; Qian, C.; Liu, X. W. Real-Time Plasmonic Imaging of the Compositional Evolution of Single Nanoparticles in Electrochemical Reactions. *Nano Lett.***2022**, *22* (11), 4383–4391 (ref (359)). Copyright 2022 American Chemical Society. (D) Monitoring object dissolution. DFM setup used to probe single coccolitophore calcification degree by dissolution using electrogenerated H^+^. Reproduced from Opto-Electrochemical Dissolution Reveals Coccolith Calcium Carbonate Content, Yang, M.; Batchelor-McAuley, C.; Barton, S.; Rickaby, R. E. M.; Bouman, H. A.; Compton, R. G. *Angew. Chem. Int. Ed.***2021**, Vol. 60, Issue 38 (ref (404)) under CC-BY 4.0 license. (E) Monitoring gas evolution reactions. Schematic and DFM images, at different potential values, of IrO_2_ NPs hopping, induced by oxygen nanobubbles formation at the interface between NPs and electrode. Reproduced from Wang, J. G.; Zhang, L.; Xie, J.; Weizmann, Y.; Li, D.; Li, J. Single Particle Hopping as an Indicator for Evaluating Electrocatalysts. *Nano Lett.***2022**, *22* (13), 5495–5502 (ref (407)). Copyright 2022 American Chemical Society.

Figure 10A (left) presents a schematic of the strategy used to image an electrochemical system by an optical microscope. To do so, an electrochemical interface of interest is brought into the field of view of a microscope objective. This lens collects the light coming from the interface and sends it through to a photodetector. If the electrochemical transformation alters the light collected by the objective, it results in a change at the detector. In wide field microscopes, a camera, made of millions of detectors, captures instantaneously a full field image (millions of pixels) of the interface of interest. Confocal optical microscopy would rather focus on the photons coming from a single submicrometer region, defining the pixel of the image, and the full field imaging of the interface requires moving the observation region as in SPMs. The interest in imaging electrochemical processes by wide-field optical microscopes is two-fold. First, it is possible to capture a single image snapshot (typically 50 × 50 μm^2^) containing thousands of pixels (localized optical information) within a millisecond time scale. Second, the imaging process is usually not perturbing to the electrochemical process and does not require accounting for invasive contributions (electrochemical, geometrical confinement, or displacement) of a nearby local electrochemical probe. Thus, optical microscopy provides a unique means to observe *operando*, and at high throughput, electrochemical processes in conditions near to those of real electrochemical systems.

However, imaging electrochemical processes with an optical wide-field microscope involves catching the optically visible footprint of the result/consequence of an electrochemical reaction. Thus, the local optical information collected at each pixel of the optical image carries (and may be converted) into local electrochemically relevant information.

Quantitative electrochemical imaging from optical microscopy is therefore demanding, as the data incorporate the chemical complexity of the systems and also requires the appropriate analysis or conversion of the optical image. As a consequence of the former point, optical microscopy has first tackled model (or simpler) systems and made use of basic light detection principles. Some of these are recalled in Figure 10A (right); in essence, objects are imaged from the collection of light they emit or light they absorb or scatter. Emission-based microscopies, such as fluorescence (FM),^22,25,26^ electrochemical luminescence (ECLM),^325^ or Raman-based (RM) microscopies, usually require labeled tracers (fluorescent or Raman active molecules or quantum dots). Label-free microscopies are sensitive to the local change in refractive index associated with an electrochemical reaction. This was mostly addressed from the change in absorbance for an electrochromic object, or change in light extinction, for example for plasmonic NPs. For uncolored dielectric objects, light variations in the refractive index can also be probed by interferometric imaging mode, as exploited, for example, by the interferometric scattering (iSCAT) or other equivalent microscopes.^326−328^

#### Methodologies for Quantitative Image Analysis

The image formation requires a significant change of the local optical properties relative to the background level. Sensitive detection and imaging then mostly rely on the ability to image a system with the lowest background level.

##### Reaching Single-Object Detection by Superlocalization

The concept of superlocalization (enabling super-resolution imaging) consists of resolving the center of mass or centroid, by a Gaussian fit procedure, of the optical feature representing the imaged object. Single fluorescent molecular probe sensitivity is reached by FM, in what is known as single-molecule localization microscopy (SMLM).^22^ SMLM was used to monitor the dynamics of molecular adsorption and electrochemical or catalytic processes at various nanoscale interfaces, such as NPs or nanobubbles.^329−335^

Removal of the background incident light is usually achieved through oblique incidence illumination (e.g., in dark-field illumination). Total internal reflection (TIR) incidence allows observation, with higher sensitivity, of the few 200–300 nm adjacent to the illuminated interface. Different single-object studies rely on superlocalizing the position of single objects in this illuminated region. TIR illumination is also used with label-free microscopies. It enables the observation of any type of nanoobject deposited on or near a thin plasmonic Au electrode in the surface plasmon resonance microscopy (SPRM) configuration.^324,336,337^ At nonplasmonic (transparent) electrodes, TIR illumination allows tracking the growth of nanodendrites with nanometer spatial resolution.^338,339^ It has particularly been used to image the formation of EDL at individual NPs.^327^

##### Interference Based Techniques

There has been increased interest in interferometric-based strategies. They are exploited in different reflection-based microscopy configurations, the most popular being the iSCAT.^326,328^ The visualization principle relies, as sketched in Figure 10A (right), on the collection by the photodetector of the interference between the light scattered by the object of interest and the beam reflected by the planar (electrode–electrolyte) interface where it is standing. The optical images consist of interference patterns, and the objects can appear either dark or bright contrasted compared to the background (i.e., the reflection from the naked interface). Although the different contrasts in the images make their interpretation less straightforward, this configuration pushes the limit of detection by several orders of magnitude compared to direct (dark-field for example) imaging.

##### ECLM: A Near-Surface Microscopy without Light Source

Recent efforts have sought to push the detection limit of ECLM, since the light emission event is triggered by an electrochemical reaction and therefore does not require an illuminating light source. ECLM is therefore operated under almost ideal dark conditions, though under the constraining conditions of producing reactive species at an electrode under strongly oxidative (or reductive) potentials.^325^ As it relies on the reaction-transport of electrogenerated species, ECLM is pertinent to imaging the adhesion sites of single biological cells.^340−345^ Interferometric measurements can also be implemented.^346^ Ultrasensitive cameras are now capable of reaching the ultimate detection of single photons. ECLM is then ideal for imaging objects or reactions nearby the electrode region with single-photon sensitivity.^344,347^

#### Converting Local Optical Information into an Electrochemically Relevant Signal

The most straightforward information in an optical image is the object position (from the localization procedure) along its 2D or 3D trajectory toward a polarized electrode, which correlates to the electrochemical current in electrochemical nanoimpact studies.^348−352^ The changes in optical signature during an electrochemical experiment carry valuable chemical information, complementary to the electrochemical signal. In particular, characteristic times (duration, onset, etc.) of the optical signature variations can be associated and correlated to different (electro)chemical transformations (conversion, dissolution, growth, etc.; see below). Several strategies have also been proposed, to convert the optical signature variation quantitatively into an optically inferred electrochemical curve, such as an optical-voltammogram.^23,323,353−356^ Light absorbance, or the extinction wavelength (for plasmonic NPs), is usually proportional to a local concentration or amount of electrochemically transformed material, i.e., an electrochemical charge.^357^ The electrochemical current then often tracks the time derivative of local optical transients. For more complex situations, optical models have been developed to simulate the change in optical intensity associated with the electrogenerated growth of NPs,^353^ the conversion of NPs,^358,359^ or nanobubbles^360^ from electrodes or electrocatalytic processes at NPs.

#### Computing and Automatized Image Analysis

Another means to extract quantitative information from optical images is to compare them to colocated correlative complementary higher-resolution microscopies, such as electron microscopy (or energy dispersive X-ray spectroscopy, EDX) images. Even if such correlative imaging is obtained *ex situ*, it provides a complementary imaging (sizing, structure, etc.) for a large number of imaged objects from which structure–optical response relationships can be obtained. A large number of objects often need to be analyzed, and automated image analysis routines can be used.^356^ These routines, often employing machine learning strategies, are now able to compare/recognize automatically images obtained from different microscopies, in order to find the same features, and classify them according to their activity, shape, or structure.

### Imaging Single Events

#### A Complement to Single Nanoobject Electrochemistry

##### Tracking Trajectories, Seeing the Transformation

The renewed interest in optical microscopy in electrochemistry is intimately related to the expansion of the field of single-entity electrochemistry.^6,28,361^ This area grew from the meticulous examination of electrochemical transients associated with the reactive collision of individual nanoobjects onto an electrode; optical observation was rapidly implemented not only to see these individual collision events but also to provide insightful complementary information. Following the seminal works of Crooks^362^ and Tao,^363^ two types of information can be gathered. First, high-throughput, wide-field-of-view imaging of the electrode region allows observation of where and how nanoobjects encounter the electrode. Spatiotemporal localization of a wide variety of individual objects (from biological objects to individual particles and molecules) can be achieved with all microscopies, including those supplemented with spectroscopic interrogation,^357,364,365^ in 2D or more recently in 3D.^366^ Second, the optical signal associated with the individual objects is scrutinized, as it provides further complementary information about the electrochemical transformation of the isolated object or in its surrounding environment.

#### Electron Transfer

##### Fluorescence Microscopy and Alternative Indirect Strategies for Imaging Nonfluorogenic Component

Emission based microscopy strategies coupled to electrochemical actuation have been successfully used over the past few years for mapping electron transfer processes with very high spatial resolution. This approach reveals the heterogeneity of electrode reactivity. To enable the imaging of electron transfer at nonfluorogenic species, Zhang et al. proposed a method using a bipolar electrode system to directly couple a conventional electrochemical (e.g., oxidation) reaction on one pole of the bipolar electrode to the reverse electrochemical (e.g., reduction) conversion of a fluorogenic species on the opposite pole.^367^ By observing optically the opposite pole electrode surface by FM, one can obtain an image of the reaction of interest of the nonfluorogenic species. The imaging strategy can be coupled with both electroactive or a pH-sensitive fluorogenic species that fluoresces upon (electro)chemical transformation.^368,369^

As a single-electron transfer can result in multiple photon emission, transducing electrochemical events into light emission can dramatically enhance the detection sensitivity up to single electron transfer events. Using an ECL reaction at the detection pole allows, in principle, higher sensitivity (lower noise).^370^ The strategy can be employed to image a wide range of electrochemical processes or serve as an indirect quantitative fluorescence sensor for nonfluorogenic redox labels for cellular biomarkers^371^ or respiration.^372^ The pixel in the image is given by the size and density of the bipolar electrode arranged in arrays. Resolution at the micrometer scale is sufficient to image biomarkers bound at cell membranes,^373^ while the use of nanoelectrode arrays improves resolution.^374^

##### Single-Molecule Fluorescence Microscopy for Imaging Single-Electron Transfer

ECL microscopy was used at the level of single-molecule or single-event imaging. The advantages of this approach are the near-zero optical noise level imaging and the absence of photobleaching usually encountered in single-molecule fluorescence microscopy studies. Single-molecule sensitivity allowed detection and imaging of the position of single proteins at the cell membrane.^375^ Single-molecule ECL imaging also considerably increases the spatial resolution of ECLM down to a few hundred nanometers by applying the concepts of super-resolution microscopy. This approach enabled the facet and defect-dependent electrochemical activity of Au nanorod and nanoplates to be determined.^376^

Single-photon ECLM was employed to resolve spatially and temporally single tris(2,2′-bipyridine)ruthenium(II) complex, Ru(bpy)_3_^2+^, oxidation reaction at electrodes. By using large excess of coreactant to ensure the excited state generation (and light emission) is confined near the electrode surface, and collecting and counting single photons over time resulting from single reaction events, Dong et al. implemented such dynamic single-photon counting to draw super-resolved images of electrodes and cells.^344^ The strategy was used to map the Ru(bpy)_3_^2+^ oxidation kinetics, analyzed to produce the standard rate constant, *k*_0_, and the standard potential, *E*^0^, over a catalytic gold microplate catalyst surface. Super-resolution images of these descriptors are provided in Figure 10B.^347^ This study revealed that the more active sites, characterized by higher reaction rate constants, are located at the edges of the catalyst. By comparing the TOF maps, constructed at different potentials, they also highlighted the formation of Au oxide on the catalysts at higher overpotential, resulting in a decrease in activity.

#### Probing Concentration Profiles

The heterogeneity of charge transfer is not just restricted to the polarized electrode or its electroactive domains, but it propagates, through mass transfer, within diffusion distances. Optical microscopy methodologies have also been developed to map the transport of optically active species involved in electrochemical reactions. It is indeed a unique, simple instrument enabling imaging *operando* molecular transport from/to electrode regions. The most commonly imaged targets are the electroactive species engaged in the reaction: fluorogenic electroactive or pH sensitive species, light absorbing or refracting reaction products or substrates, and scattering NPs. Recent strategies are devoted to the transport of the electrolyte itself (ion fluxes) during the electrode reaction: from fluorogenic or Raman-active electrolyte ions to fluorogenic electroinactive molecules or particles, which are used to monitor the accessibility or flow of the solvent to porous electrodes.

3D mapping is also possible through confocal imaging (under pseudo-steady-state electrochemical conditions),^377−379^ illustrated recently from the 3D diffusion layer build-up during enzymatic reactions.^380^ The instruments are sufficiently mature to tackle the transport of active species in real energy storage or conversion systems, such as porous electrodes^381^ for electrolyzers or batteries^382−384^ under operation.

Refractive based microscopy was recently used to image the local transport of Li^+^ ions during (dis)charging currents within individual micrometric electroactive LiCo oxide or NbW oxide microparticles.^382,383^ Merryweather et al. suggested from the dynamic analysis of these ion propagations that the charge and discharge processes do not follow the same trajectory within the particle. These differences are discussed based on the difference in conductivities in each state of the particle, which is then manifested locally.^382^ The method was extended to imaging in the presence of the usual electrode components (polymer binder and carbon paste) and developed to enable 3D probing inside the particles using confocal visualization.^384^ Complemented with 2-photon fluorescence excitation, the local distribution of the LiPF_6_ electrolyte could be imaged around the particles, revealing inhomogeneous electrolyte diffusion as a result of the geometry and porosity of the carbon/binder matrix surrounding the particles.

#### Conversion

As the electrochemical transformation of a single entity usually provokes a significant modification of its intrinsic optical properties, optical imaging can be employed to follow the conversion process, to identify reaction intermediate, and to decipher mechanistic routes. The conversion of electrochromic NPs can be monitored from local absorbance imaging. For instance, Evans et al. monitored and quantified the conversion of single WO_3_ NPs, i.e., indirectly the lithium ion intercalation process,^385^ via changes in the optical density. From the experiments, it was concluded that pseudocapacitive charge storage depended on the NP morphology. The same methodology was also applied in a comprehensive series of studies from Wang’s group on the (indirect) visualization of mass transport of potassium ions inside single Prussian blue particles during their reversible electro-chromic conversion to Prussian white.^354,355,386^ Optical data suggested a less intuitive shell-to-core transformation mechanism^355^ and evidenced partial conversion due to an inactive zone inside some of the studied particles.^386^ The same material was further investigated by coupling optical visualization to EIS to probe the depth of the surface charging layer during the same redox conversion.^387^

Optical phase retrieval from SPRM allows a quantitative direct evaluation of refractive index at the single-NP level. This strategy also brings selectivity to label-free imaging for discriminating between different types of chemistry of NPs (Figure 10C).^359^ The approach was also used to discriminate between surface charging and compositional evolution during the electrochemical conversion of Ag into AgCl NPs that was previously imaged by interferometric reflection microscopy.^351,358^ Again, the conversion does not follow the same intermediates and dynamics during oxidation or reduction. The reduction of the less conductive AgCl NP starts with heterogeneous surface inclusion before a core–shell transformation operated. As the visualization also provided spatial information, the NPs were shown to move on the electrode during multistep charge injection of the electrochemical conversion. It was hypothesized that the local halide ions released during the conversion propelled the NPs.^358^

#### Growth and Dissolution

Electrochemical reaction can lead to the creation of nanostructures that can be imaged at the early stage of their formation by optical microscopies.^388^ A case study is the electrochemical deposition of noble metals such as Ag, Au, or Cu, for which the nucleation and growth mechanisms are still under intensive investigation. Dark-field spectroscopic microscopy (DFM) detected the facet-dependent deposition of atomic layers of silver on single gold NPs.^357^ Visualizing *operando* deposition processes also gives access to the evolution of the surface nucleation density.^389−391^ As the local scattering intensity is linked to the NP volume, it also allows sizing single NPs over time and the measurement of their growth dynamics.^353,390−393^ By differentiating the NP volume fluctuation, one can further calculate an “optical current” related to the growth of single NPs.^353,391^

Optical microscopy has been employed for imaging the electrodeposition of more complex materials such as Ni,^356,391^ Co,^392^ Li,^394,395^ Mg,^396^ and Zn^338,339^ as metal and/or as metal oxides and hydroxides (Ni,^391^ Mn,^397^ or Zn^397^). These studies have implications in electrocatalysis and battery processes, as well as for studying the electrocrystallization of metal oxides for energy conversion and storage. For instance, optical microscopy, employed as an “optical microbalance”, evidenced the direct influence of zinc hydroxide sulfate precipitation/dissolution during the dissolution/deposition of the MnO_2_ electrode associated with its discharge/charge in an aqueous Zn–MnO_2_ battery environment.^397^

Optical visualization can also be a “helping hand” in the elaboration of heteronanostructures, as exemplified by the monitoring of the electrochemically assisted deposition of shells of various composition around Au NPs. These studies take advantage of the sensitivity of the localized surface plasmon resonance spectrum of the NP to alteration of its surface composition. The electrodeposition is then tracked from the change in color of the NP observed by DFM. It was used to track amalgamation of Au with Hg,^398−400^ the electrodeposition of mono- or bimetallic shells (Ag^357^ or Pt, Pd, Rh, PtPd, and PdRh^401^) or semiconductor shells (CdS, CdSe, and ZnS) around Au NPs.^402^

Optical monitoring of the dissolution kinetics of single entities by the same methodology is also possible. The electrodissolution of single Ag NPs continues to be actively investigated.^24,364,365,403^ One way of analyzing single NP dissolution kinetics from optical images consists of evaluating the characteristic duration of the decrease of scattered light with time by DFM. The same strategy was used (Figure 10D) to image the dissolution of single micrometer-sized marine entities of calcium carbonate (coccoliths) triggered by the electro-generation of acid.^404,405^ This opto-electrochemical titration, governed by surface reaction limitations, allowed reconstruction in 3D of the entity volume based on dissolution kinetics and determination of the initial CaCO_3_ mass, as well as to investigate the effect of some adsorbates (Mg^2+^) on the dissolution rate. The strategy is sound as a means of evaluating the CO_2_ storage capacity of biomineralizing algae. A key general feature, when studying dissolution/growth at microscopic single entities, is that mass transport (diffusion) is well-defined and high, thus enabling the measurement of fast kinetics with simple platforms.^406^

#### Catalysis and Motion

The accumulation of products at the vicinity of a nanoscale electro-catalyst sometimes provokes a change in the local refractive index of the surrounding medium that can further be detected by optical microscopy.^363^

Electrocatalytic reactions such as hydrogen or oxygen evolution reaction often produce nanobubbles at the electrode surface that nucleate and grow once the local environment becomes saturated by gas molecules (as described in Phase Formation). It has been proposed that those bubbles act as nanoreporters of catalytic activity, and therefore, it has been argued that mapping these nucleation sites reveals the most active region of the electrode.^408^ Optical microscopies are ideal for monitoring dynamically and under operating conditions the dynamics of formation and growth of nanobubbles on surfaces.^409,410^ Nanobubbles were imaged by single-molecule fluorescence microscopy using fluorescent probes that are trapped at the liquid–gas interface,^333^ even forming molecular aggregates.^335^ Using this methodology, the Zhang group imaged the hydrogen spillover occurring at gold nanoplates supported on ITO electrodes during OER^331^ and the delayed production of H_2_ nanobubbles at AuPd electrode during HER that could be explained by the ability of palladium to store hydrogen.^411^

An alternative for imaging nanobubbles is to rely on sufficiently sensitive scattering-based microscopy. The formation of nanobubbles is directly monitored at NPs by interferometric or DF microscopies^360,407,412^ or at the step edge of graphene by SPRM.^413^ These microscopies are meant as a tool to investigate gas evolution mechanisms. For example, a graphene electrode polarized at negative potential is shown to produce O_2_ gas bubbles suggesting the graphene acts, during ORR, as a superoxidedismutase.^413^ The growth of the electrogenerated gas bubble can also be tracked optically. This growth is revealed as a transient increase in the optical intensity, confirmed by optical modeling.^360^ Coupled with superlocalization, the nanobubbles were shown to sometimes disconnect the NPs from the electrolyte while they continued growing, revealing the existence of a possible cross-talk among the NPs.^360^ At high gas evolution rate, images show bubbles fading away with time, which, taking advantage of the NP-distance versus scattering intensity dependence in SPRM, is attributed to the lift of the nanocatalyst above the bubble. Wang et al. tracked the vertical motion of the nanocatalysts (IrO_2_, doped Ca_3_Co_4_O_9_, and Pt-decorated C_3_N_4_ nanosheets) during OER caused by the formation of O_2_ nanobubbles between the electrode and the catalysts and further used this hopping behavior as an indicator of catalytic activity (Figure 10E).^407^ By doing so, they highlighted the concept of motion–activity relationships that should be taken into account in many catalytic reactions. Thus, optical microscopy provides direct evidence for the movement of NPs during gas evolution, which can only be inferred from electrochemistry alone, such as current–time traces in NP impact experiments.^414^

Motion is an important characteristic of nanobubbles. At even higher gas evolution rates nanobubble formation and detachment from the electrode is detected through blinking events in DFM.^415^ Blinking fluorescence events also allow reporting by ECLM of single-nanobubble formation and motion.^416^ Motion along the electrode plane during the nanobubble formation or dissolution is generally probed by superlocalization principles.^303,409,417,418^ It is related to the weak adhesion of the bubble on the electrode surface until the contact line is pinned at strong adsorption sites.

The question of motion of nanoobjects near an electrode is also a central question in understanding the processes involved in electrochemical collision studies.^419^ Various optical microscopy configuration are able to track the trajectory of individual nanoobjects toward their adsorption/reaction at an electrode,^376^ also allowing the imaging of the rotation and reorientation of individual graphene nanoplatelets during nanoimpacts on a microelectrode.^352,420^

### Competing Processes

The *operando* visualization of different optical contrasts or features at a polarized interface allows analysis of the possibility of parallel or competing electrochemical processes. Beyond the competition between mass transfer to, and reaction at, the electrode, or the observation of higher current densities at electrode edges, an electrochemical reaction is linked to the local heterogeneous activity of the electrode, as described in detail in High-Throughput Scanning Electrochemical Cell Microscopy. Consequently, different electrode reactions may occur at distinct locations at the electrode, at the same electrode potential, leading to variation in the local products. To identify competitive product formation (and to evaluate Faradaic efficiency), it is necessary to complement the electrochemical current–potential curve by complementary characterizations. Optical microscopy can provide a unique mechanistic tool when different reaction pathways are revealed as distinct optical footprints. Optical imaging evidences the physical (or chemical) alteration of the electrode during, or in relation to, the electrochemical process or allows evaluation of the competition between chemical routes in electrosynthesis.

#### Electrochemistry versus Physical Transformation

**Figure 11:**
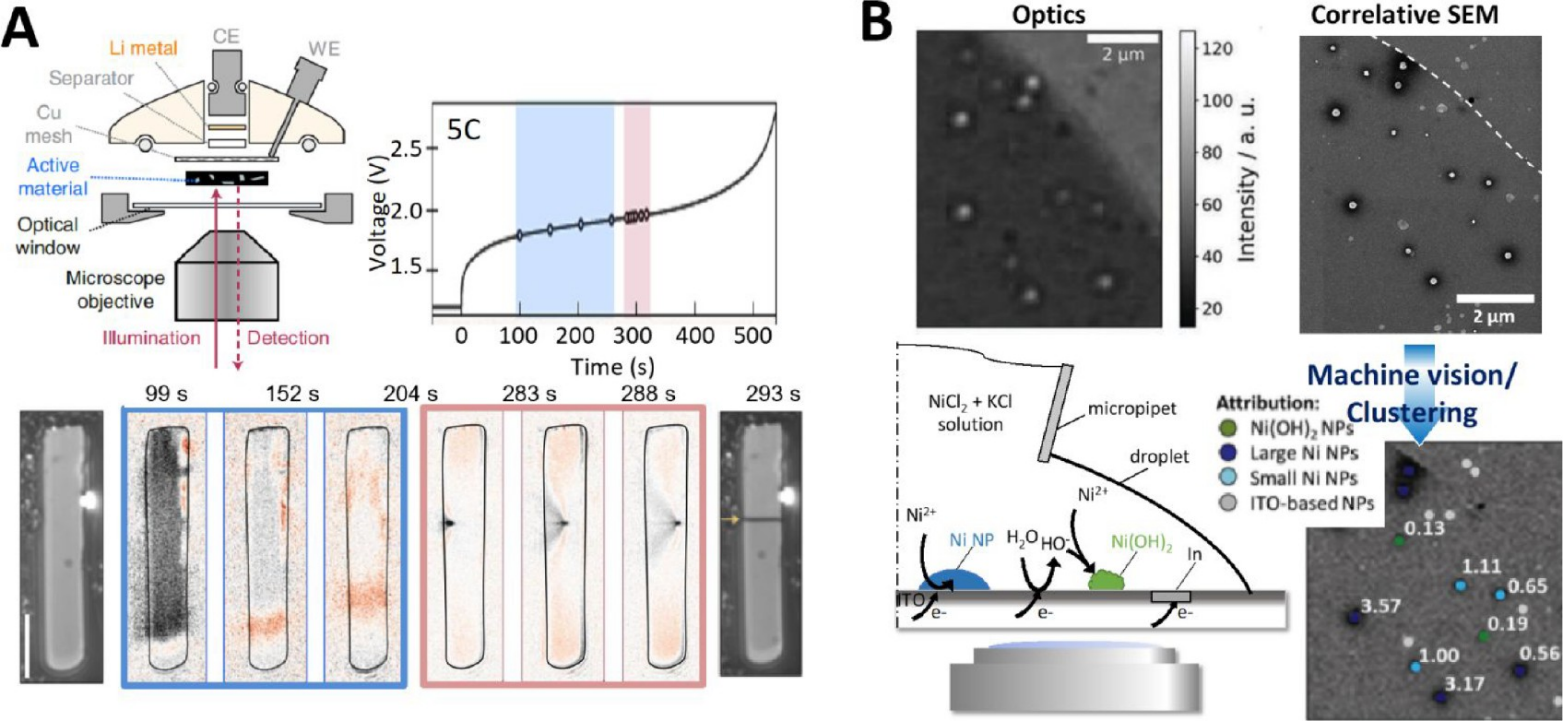
(A) Probing Li^+^ ion diffusion during niobium–tungsten particle charging. Schematic of the optical setup used to probe *operando* the cell voltage charge ion due to migration, leading to particle cracking. The fracture propagation can be observed due to optical scattering images at 283 s from the beginning of polarization. Reprinted by permission from Macmillan Publishers Ltd.: Nature Materials, Merryweather, A. J.; Jacquet, Q.; Emge, S. P.; Schnedermann, C.; Rao, A.; Gray, C. P. *Nat. Mater.***2022**, *21* (11), 1306–1313 (ref (383)). Copyright 2022. (B) Correlative multimicroscopy approach coupled with machine vision algorithm for probing the electrodeposition of Ni-based NPs. Reproduced from Bridging the Gap between Single Nanoparticle Imaging and Global Electrochemical Response by Correlative Microscopy Assisted By Machine Vision, Godeffroy, L.; Lemineur, J. F.; Shkirskiy, V.; Miranda Vieira, M.; Noel, J. M.; Kanoufi, F. *Small Methods*, Vol. 6, Issue 9 (ref (356)). Copyright 2022 Wiley.

The electrochemical conversion or processes of ion insertion during energy storage material charge or discharge can be accompanied by considerable physical transformation. Relative volume expansion/shrinking as low as a few percent can be probed from the superlocalization of individual microparticle edges during its conversion (e.g., cobalt oxide)^421^ or Li^+^ ion intercalation (in NbW oxide).^383^ Dynamic imaging of the local state of charge (Li^+^ ion front) in an NbW oxide microparticle during lithiation and delithiation reveals phase separations within the particle. Figure 11A presents a time series of differential optical contrast images during the delithiation of an NbW oxide microrod. Initially, the particle darkening (delithiation) is rather homogeneous over the particle, except for the white lower part of the rod from which a front of different delithiation rate (red domains) propagates toward the upper end of the rod. This heterogeneous state of particle charge (observed during both charge and discharge) is related to the dependence of Li^+^ diffusion coefficient on the state of charge. Phase separation from charging the particle at extreme rates of delithiation (5–20 times the nominal charging rate) can even lead to particle dislocation or rupture, also detected optically from the appearance of the dark dot at 283 s and propagating later from the left to the right of the particle in Figure 11A.^383^

#### Competing Electrochemical Reactions

A typical example of the use of optical microscopy to investigate parallel or competing chemical reactions is the monitoring of electrochemical processes occurring within the potential regions associated with electrolyte discharge, as illustrated by the electrodeposition of Ni NPs from Ni^2+^ ion solution in neutral pH.^356,391^ Owing to the negative potential needed for the reduction of the Ni^2+^ ions, the electrodeposition of metallic Ni NP competes with the electrogeneration of OH^–^ from water reduction at the electrode. The latter results in the precipitation of Ni(OH)_2_ nanocrystals. The competition between metallic Ni and Ni(OH)_2_ NP formation is distinguished by interference reflection microscopy: the former appear as bright optical features, while the latter appear as dark-contrasted features.^391^ The use of electrolyte microdroplets, produced from micropipettes, allows probing the electrochemical reaction over different regions of the electrode (a transparent ITO surface). Interestingly, the results strongly depend on the local electroactivity of the ITO electrode itself, as either a mixture of Ni and Ni(OH)_2_, or a single population of Ni(OH)_2_ NPs can be formed. The contribution of each competitive route also depends on the electrode potential (and Ni^2+^ ion concentration) and can be identified by comparing the dynamic growth of each nanomaterial population. In essence, identifying competitive paths requires analysis of optical images of objects or domains presenting different chemical structures. *Operando* optical monitoring needs to be compared with multiple complementary and correlative microscopies of the electrode at identical locations. As illustrated in Figure 11B, SEM images were correlated with the *operando* optical images. A supervised machine learning analysis and feature recognition in the different sets of images enabled identification and classification by size and chemistry of the different electrogenerated nanoobjects.^356^

Confining the electrochemical reaction with micropipettes, in an SECCM type experiment, within microsized regions of the electrode reveals the great heterogeneity of the electroactivity of ITO for metal electrogeneration versus water reduction. Indeed, a third population of NPs was also found corresponding to the reduction of ITO into metallic In NPs. Replacing the Ni^2+^ solution by 5 mM H_2_SO_4_ allowed inspection of the hydrogen evolution reaction at the ITO surface and revealed its competition with In metal formation.^303^ Optically, In NPs and H_2_ nanobubbles appear with similar contrast within the same negative potential region and disappear upon backward (positive) polarization. Their identification within optical images is obtained by superlocalizing the nucleation and mobility of the different objects on the electrode. Over successive potential cycles the In NPs are nucleated and (reversibly from the optical point of view) reoxidized at the same location. In contrast, the H_2_ nanobubbles nucleate at stochastic sites over repeated cycles. Moreover, the growth/dissolution of bubbles on the electrode is also detected as a mobile optical feature, which highlights the pinning of the bubble at electrode surface defects. It is noteworthy that In_2_O_3_ reduction and H_2_ generation compete for H^+^ ions, resulting in segregated regions of micrometric size for each population over the same electrode.

### One versus Many

#### Seeing Collective Behaviors

The nucleation/growth of new phases, as in electrodeposition or electrocrystallization processes, is often associated with collective behaviors. These can be understood from high-throughput optical microscopy that allows observation of individual entities and the ensemble. Optical observation revealed intrinsic growth kinetics of each individual NP within an ensemble, along with apparent progressive growth associated with the broad distribution of NP nucleation or onset times.^353,389−391,422^ Label-free optical microscopies (dark field, interference reflection, or wide-field SPR) are now able to image NP densities up to 10^9^ NPs per cm^2^. They have allowed imaging of the dynamics of NPs, from onset, during the electrodeposition of Ag,^353,389,422^ Cu,^390^ or Ni (vs Ni(OH)_2_) NPs,^391^ or the electrocrystallization of CaCO_3_ particles.^231^

Collective behaviors are related to the overlapping of the diffusion (concentration boundary) layers to sustain NP growth. Chemical communication between NPs, from overlapping of diffusion fields, could be evaluated from the distribution of sizes of the Voronoi (elemental) cell occupied by each NP during their growth.^389^ When there is significant reactant depletion, the nucleation of new NPs is delayed in regions near existing ones and the growth is slowed down.^390^

The spatial distribution of NPs on the surface is therefore strongly related to the diffusion field of electrogenerated species. This is particularly demonstrated in multicomponent deposition processes such as the electrocrystallization of CaCO_3_ nano/microparticles.^231^ The formation of such crystals is due to the local alkalization of the Ca^2+^-containing electrolyte owing to OH^–^ electrogeneration during ORR. The reaction was monitored by interference reflection microscopy, watching the meniscus of an SECCM microscale-sized electrochemical cell (as described in Phase Formation). Interestingly, this hyphenated configuration allows inspection of electrode transformations with submeniscus resolution down to 500 nm meniscus diameter. In tens of micrometer-diameter menisci, the CaCO_3_ crystals precipitate first near the edges of the meniscus, then progressively nucleate toward its center. The spatiotemporal distribution of CaCO_3_ crystals was rationalized, from simulation, from the interplay between the pipet mass transport, the oxygen reduction rate, and the CO_2_ and O_2_ transport across the air/meniscus interface.

#### How to Access Missing Pieces of Information

The spatiotemporal distribution of NP nucleation/growth events discussed above could not be anticipated from the sole inspection of the ensemble-averaged electrochemical response. When individual object or domain activity is imaged optically, the sum of the optically inferred activity of all the individual domains can be compared to the ensemble electrochemical response. This optical-electrochemical correlative strategy was achieved in a number of situations, most recently for the electrodeposition of Ag^353,389,423^ and Ni NPs,^356^ the electrogeneration of H_2_ nanobubbles,^360,407^ the charging/discharging of the MnO_2_ electrode for Zn/MnO_2_ batteries,^356^ and graphene layer oxidation and reduction.^424^

As also highlighted in SECCM multiscale measurements, sometimes, the optically inferred information does not match the macroscopic electrochemical behaviors. For example, the cyclic voltammogram for the electrodeposition of a single Ni NP can be inferred from the optical monitoring presented in Figure 11B,^356^ but the sum of all the individual voltammograms shows a mismatch in charge or electrochemical current overall. An interpretation is that the reduction of Ni^2+^ and formation of Ni is accompanied by the electrocatalysis of water reduction at the Ni NP. Similarly, the reduction of MnO_2_ thin layers during discharge is accompanied by a lower (optically inferred) dissolution rate than expected based on Faraday’s law. This mismatch suggests the MnO_2_ reduction results in a partial dissolution of the MnO_2_ layer accompanied by the precipitation of a foreign hydroxide layer from the electrolyte. This solid electrolyte interphase formation, which is detected optically, may impact the overall cyclability of the electrode material.^397^

#### Hyphenation with Local Complementary Information

Since the role of individual entities is intimately related to that of the ensemble, multimicroscopy identical location imaging approaches are essential as we have described herein. As also described in the SECCM section (Phase Formation), such developments are important when seeking to understand electrochemical systems involving the electrocatalysis of complex reactions that yield different products and intermediates, together with structural reconstruction of the catalysts.

Obviously, this requires large and multidimensional data sets from which trend lines, correlation, and classification becomes less straightforward for a single operator. The handling of large data sets and their analysis is now met by data science and machine learning approaches, which are also impacting the automatized analyses of images. Recent reviews discuss the introduction of machine learning approaches for identifying the different components of an electrode material in batteries.^425,426^ The identification of Ni versus Ni(OH)_2_ NPs formation used a machine learning clustering based on optical, SEM, and EDX images colocalized by automatized pattern recognition algorithms.^356^ Machine learning strategies are also intensively applied in optical microscopy to make these tools quantitative.^427,428^

## Other Electrochemical and Electronic High-Throughput Imaging Techniques

In closing this article, we briefly mention some additional local electrochemical imaging techniques that are currently being used to examine electrochemical systems. They require highly expert users but are ripe for high-throughput nanoscale measurements. In the field of local electrochemical probes, SPMs, such as AFM and STM, combined with electrochemistry have probably been the most widely used. In particular, it is important to note the possibility of coupling these imaging methods to different local electrochemical (SECM, SICM, or SECCM)^5^ or spectroscopic (TERS)^429^ probes. In the context of high-throughput imaging, while maintaining atomic or nanometer-scale image resolution, it is possible to image surfaces under electrochemical operation at a rate of 30 images per second (i.e., almost 3 orders of magnitude faster than a conventional SPM), with so-called video-rate imaging-STM or AFM.^430^ This approach has enabled studies of different single-crystal electrode surfaces and associated surface dynamic changes for model systems. Examples include the formation of surface adsorbates and their surface diffusion (or migration) under polarization, e.g., sulfur adsorbates on Br-coated Ag(100),^431^ or on Br- or Cl-coated Cu(100),^432^ or CO onto Pt(111).^433,434^ The strategy is currently extended to more complex situations illustrated by the observation with video-AFM of (i) the dynamics of nanobubbles on electrodes during water electrolysis^435^ or (ii) a wide variety of dynamic restructurings of model Cu(100) catalyst electrode surface during electrochemical CO_2_ reduction.^436^ In the latter case, under open-circuit potential, an epitaxial oxide layer is formed atop the Cu electrode. Under moderate reduction potentials the Cu catalyst is transformed from smoothly curved surface to rectangular terraced surface, while at higher cathodic potentials the density of step edges (uncoordinated Cu sites) is drastically increased.^436^ Such measurements can be linked effectively to SECCM-EBSD^236^ (Popular Redox Reactions and Electrode Materials and Corrosion) to provide a holistic view of structure–activity dynamics.

**Figure 12:**
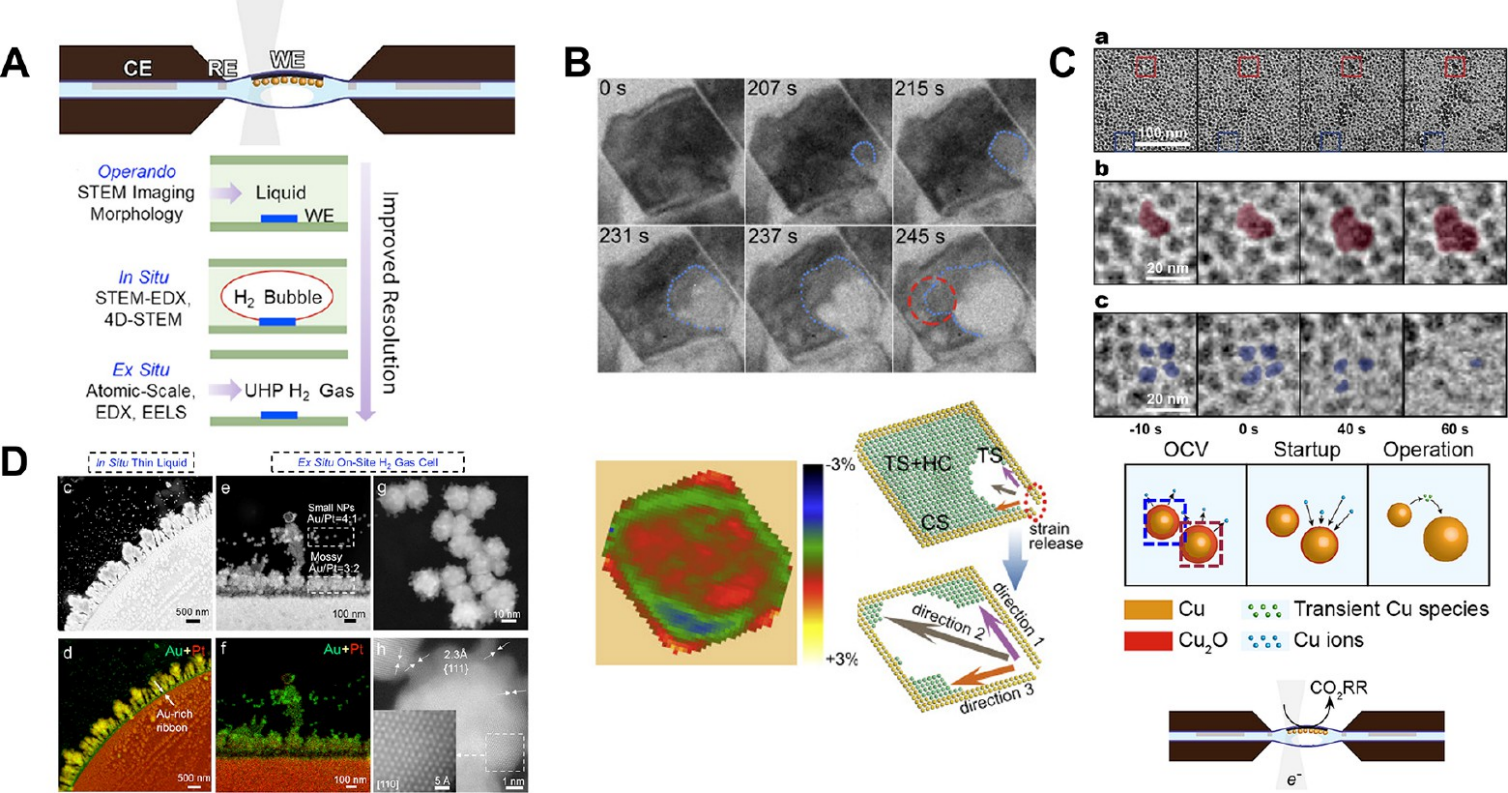
*Operando* electrochemistry in LC-TEM. (A) Schematics of the Si_*x*_N LC for operandi LC-TEM with examples of the characterizations and imaging resolution that can be achieved with identical location *operando*, *in situ* (with large gas bubble formed), or *ex situ*. Reproduced from Yang, Y.; Shao, Y.-T.; Lu, X.; Yang, Y.; Ko, H.-Y.; DiStasio, R. A., Jr.; DiSalvo, F. J.; Muller, D. A.; Abruña, H. D. *J. Am. Chem. Soc.***2022**, *144* (34), 15698–15708 (ref (449)). Copyright 2022 American Chemical Society; and from Real-time Monitoring Reveals Dissolution/Redeposition Mechanism in Copper Nanocatalysts during the Initial Stages of the CO2 Reduction Reaction, Vavra, J.; Shen, T.-H.; Stoian, D.; Tileli, V.; Buonsanti, R. *Angew. Chem. Int. Ed.*, Vol. 60, Issue 3 (ref (457)). Copyright 2021 Wiley. (B) Corrosion of Pd@Pt core–shell NPs during the ORR. LC-TEM successive images show the propagation of the dissolution along specific direction corresponding to regions of lower strain (colored map). Reproduced from Chem, Vol. 6 (9), Shi, F.; Gao, W.; Shan, H.; Li, F.; Xiong, Y.; Peng, J.; Xiang, Q.; Chen, W.; Tao, P.; Song, C.; et al., Strain-Induced Corrosion Kinetics at Nanoscale Are Revealed in Liquid: Enabling Control of Corrosion Dynamics of Electrocatalysis, pp. 2257–2271 (ref (453)). Copyright 2020, with permission from Elsevier. (C) Electrochemical Ostwald ripening during eCO_2_RR at Cu NPs. The NPs in the red square are growing during the reduction step while the smaller ones (blue square) are dissolved into Cu ions. Reproduced from Real-time Monitoring Reveals Dissolution/Redeposition Mechanism in Copper Nanocatalysts during the Initial Stages of the CO_2_ Reduction Reaction, Vavra, J.; Shen, T.-H.; Stoian, D.; Tileli, V.; Buonsanti, R. *Angew. Chem. Int. Ed.*, Vol. 60, Issue 3 (ref (457)). Copyright 2021 Wiley. (D) Reductive electrodissolution of Au/Pt nanostructures. *Operando* the formation of a Au ribbon is probed at the Au/Pt interface; higher resolution imaging reveals the detachment and formation of smaller Au NPs from the preferred reductive dissolution of Au. This *in situ* measurement configuration allows crystallographic analysis of the electrosynthesised Au nanocrystals. Reproduced from Yang, Y.; Shao, Y.-T.; Lu, X.; Yang, Y.; Ko, H.-Y.; DiStasio, R. A., Jr.; DiSalvo, F. J.; Muller, D. A.; Abruña, H. D*. J. Am. Chem. Soc.***2022**, *144* (34), 15698–15708 (ref (449)). Copyright 2022 American Chemical Society.

Recent years have witnessed an increasing number of studies and reviews^437−442^ reporting the use of *in situ* electron microscopy for electrochemical energy storage/conversion. Applications in this area have been facilitated by the mass production of liquid cell (LC) chips, thin enough to obtain high spatial resolution imaging. Commercial cells are now available, consisting of two sufficiently thin and robust SiN_*x*_ windows, 10–30 nm thick, providing good transparency to the electron beam (Figure 12A). Electrodes are deposited as thin films on the SiN_*x*_ window, and an electrolyte circulates in the cell via a fluidic system through the TEM sample holder. Since the early work on electrochemical deposition of metal NPs^443,444^ or nanodendrites,^445^ or nanoscale imaging of ion distribution during battery electrode charging,^446^ there have been some concerns raised regarding the interference of electron beam irradiation with (electro)chemical reactions, or the appropriate geometric configuration of the electrode to obtain reliable electrochemical measurements.^440,447^

These limitations have been taken into account, and LC-TEM is increasingly used as a high-throughput *operando* imaging technique.^439,448^ Its advantage is the possibility to perform multiple characterizations (Figure 12A). In addition to the *operando* imaging, developments to improve imaging *in situ* include the growth of a gas bubble to limit the liquid thickness and improve the resolution,^449^ or post-mortem or *ex situ* (after removal of the liquid) analysis at the same location to enable, *inter alia*: better resolution of morphology, detection of crystal structures by electron diffraction, to allow the analysis of deformation in nanocrystals, and chemical identification by X-ray spectroscopy. A significant focus has been toward the *operando* evaluation of the durability of materials under electrochemical conditions. LC-TEM is well-suited to evaluate structural changes of nanomaterials for battery electrodes during dis/charging (Li^450^ or Na ion batteries^451^), or of nanocatalysts under electrocatalytic conditions. In addition to dissolution kinetics (which can be probed by optical techniques described above), *in situ* TEM provides a dynamic analysis of the object structures at the nanoscale, especially during their corrosion induced by electrochemical operation. Electrocatalytic oxidations provide, in fact, aggressive conditions that can lead to the dissolution/corrosion of various nanomaterials, such as cobalt oxide NPs during the oxygen evolution reaction,^452^ or boron nitride NPs during ethanol oxidation (where SECCM was brought to bear for sample preparation, as described in Phase Formation).^271^

Wu et al. have analyzed the extent of Pd corrosion during electrocatalysis of the ORR by Pd@Pt core–shell nanoparticles (Figure 12B).^453^ It was demonstrated how Pd ion leakage is directional and related to the particle curvature and strain. The ORR is known to produce reactive oxygen species, which results in the corrosion of various nanomaterials under electrolysis conditions. It affects not only the nanocatalysts, restructuring its facets into a more stable shape, e.g., in the case of Pt–Ni nanoalloys,^454^ but can also damage the carbon support used as an electronically conducting phase.^455^

The corrosive dissolution of the catalysts also produces metallic ions, which are then prone to redeposition as metal under reductive polarization. This phenomenon was probed during eCO_2_RR at Cu-oxide nanocatalysts.^456,457^ LC-TEM is ideal for tackling such reactions, as illustrated in Figure 12C where the native Cu-oxide layer coating on NPs is restructured during the early stage of the reduction. It mostly results in the dissolution of the Cu NPs (Figure 12C, subpanel c) into soluble Cu ions, followed later by the formation (or growth) of novel Cu metal nanostructures under the reductive experimental conditions (Figure 12C, subpanel b).^456,457^

Even stronger reductive potentials are accompanied by considerable reconstruction and corrosion of metallic nanostructures. LC-TEM was used to track such cathodic-corrosion phenomenon during HER at the more stable Pt(111) surface^449,458^ to the more reactive system of single Au nanocubes deposited on Pt bulk.^449^ Both morphological and compositional changes of the Au nanostructures and of the Pt support were evidenced. The strategy presented in Figure 12A to improve the resolution enables complementary *in situ* and post-mortem imaging insights of this process with nanometer to the atomic scale resolution (Figure 12D). It particularly evidences the formation of Au–Pt alloys during the corrosion process, likely through metal hydride formation.^449^

## Conclusion

In this article we have sought to showcase considerable developments in key aspects of nanoelectrochemistry arising from advances in robust nanoscale electrochemical devices and imaging techniques that are readily implemented. Nanoscale imaging of surface dynamics now encompasses the wide variety of subjects covered by electrochemistry, from molecules to materials, from sensors to energy storage or conversion, and the intersection of the subject with the life sciences. Imaging electrochemical processes at the nanometer scale or detecting individual nano-objects (even molecules) in an electrochemical situation is becoming much more routine. The challenge, now and in the future, is to increase the throughput of knowledge through the development of strategies to both increase the volume and proportion of relevant data from an experiment (avoiding redundancy) and for the analysis of larger data sets. To this end, our article has explored efforts made in high-throughput electrochemical analysis and imaging, focused on the past few years.

While we have touched upon several SPM techniques used in electrochemistry, nanoscale electrochemical imaging (i.e., nanoscale electrochemical flux visualization and nanoscale voltammetric measurement) is most readily achieved through nanopipette-based probes, i.e., imaging by SICM and SECCM. SECCM is now used by increasing numbers of research groups in a plethora of systems impacting all fields of electrochemistry and materials. In its present form, and with further developments, including intelligent scan routines and integration with complementary (*in situ*) microscopy and spectroscopy techniques, SECCM is expected to open up fascinating understanding of energy storage (batteries, supercapacitors, *inter alia*), conversion (electrocatalysis), electrosynthesis, and corrosion at the nanoscale. The application of SECCM to these problems is attractive because these processes are intricately related in real-world nanostructured materials.

Most efforts with SICM are in relation to biological entities, but in the past few years multifunctional aspects of the technique have been developed, as evident from increasing number of strategies for surface charge mapping with SICM, the use of the technique for local delivery and its integration with complementary microscopy techniques. These developments have been driven by efforts to model and understand the SICM response, particularly with regard to nanoscale mass transport. Like SECCM, this foundation is providing opportunities for progressive use of the technique to electrochemical materials related to energy storage or conversion and to corrosion.

Nanopores currently offer sensing of individual molecules or nanoobjects at high frequency with high chemical selectivity (chemical throughput). We envision the increasing translation of nanopore developments to the SICM to enable high-throughput imaging of interfaces at high spatial and temporal resolution with high chemical sensitivity. We have described herein enhanced information from nanopores through the use of array devices and more detailed analysis of nanopore signals, as well as multifunctionality from the incorporation of additional electrochemical and optical detection strategies.

Optical microscopy strategies offer a fast, simple, and cost-effective way to monitor *operando* a wealth of electrochemical processes with nanoscale resolution, drawing on the concepts of superlocalization. While liquid-cell TEM offers higher resolution for *in situ* and *operando* visualization, there are some compromises on cell design (and mass transport), limitations on integrating the technique with complementary methods, and issues around the effect of the electron dose on the solvent and processes studied. We thus expect optical methods to become an important option for the main body of electrochemists, especially if seeking quick initial insights.

The ultimate single-molecule imaging limit is commonly achieved for fluorescent probes, but it is now at hand with label-free imaging based on promising developments in interferometric scattering microscopy. Recent advances have shifted the technique from model plasmonic systems to more complex situations, including real battery electrode materials, or to inspect further chemical complexity, such as competing chemical reactions. Furthermore, the wide field of view enables monitoring of thousands of nanoentities simultaneously, not just to resolve the behavior of single entities, but also to address the question of inter-entity communications.

The development of holistic views of nanoscale electrochemistry will increasingly make use of correlative multimicroscopy approaches. We have illustrated this aspect of nanoelectrochemistry throughout this article with recent examples that show how the level of structural, chemical, and functional (electrochemical) information on a given system is massively increased. Storing, handling, analyzing, and interpreting these data will require a shift in the field toward data science and artificial intelligence, through machine learning. The next generation of nanoscale electrochemistry and electrochemical imaging approaches will draw on automatized multi-image cross-correlation and automatized imaging tools, which will increase throughput and accelerate the discovery and rational development of electrochemical technologies so urgently needed.

## List of Abbreviations Used

2D: two-dimensional
AC: alternating current
AFM: atomic force microscopy
AI: artificial intelligence
BDD: boron-doped diamond
CG-TC: carrier generation-tip collection (SECCM method)
CVD: chemical vapor deposition
DA: dopamine
DC: direct current
DFM: dark-field (spectroscopic) microscopy
DFT: density functional theory
DOS: density of electronic states
EBSD: electron backscatter diffraction
ECLM: electrochemical luminescence microscopy
eCO_2_RR: electrochemical CO_2_ reduction reaction
EDL: electrical double layer
EDX (or EDS): energy-dispersive X-ray spectroscopy
EIS: electrochemical impedance spectroscopy
FcDM: 1,1′-ferrocenedimethanol
FEM: finite element method
FM: fluorescence microscopy
GDE: gas diffusion electrode
GND: geometrically necessary dislocation
h-BN: hexagonal boron nitride
HER: hydrogen evolution reaction
HOPG: highly oriented pyrolytic graphite
ICR: ion current rectification
iSCAT: interferometric scattering microscope
ITO: indium tin oxide
LC: liquid cell
LIBs: lithium-ion batteries
MOF: metal–organic frameworks
NP: nanoparticle
OER: oxygen evolution reaction
ORR: oxygen reduction reaction
PZC: potential of zero charge
QRCE: quasi-reference counter electrode
rGO: reduced graphene oxide
RM: Raman-based microscopy
ROS: reactive oxygen species
RRDE: rotating ring disc electrode
SECCM: scanning electrochemical cell microscopy
SECM: scanning electrochemical microscopy
SEM: scanning electron microscopy
SEPM: scanning electrochemical probe microscopy
SERS: surface-enhanced Raman spectroscopy
SHINERS: shell-isolated nanoparticles enhanced Raman spectroscopy
SICM: scanning ion conductance microscopy
SMLM: single-molecule localization microscopy
SOM: self-organized map
SPM: scanning probe microscopy
SPRM: surface plasmon resonance microscopy
STM: scanning tunnelling microscopy
TEM: transmission electron microscopy
TERS: tip-enhanced Raman scattering
TIR: total internal reflection
TMDC: transition metal dichalcogenides
TOF: turnover frequency
ToF-SIMS: time-of-flight secondary ion mass spectrometry
UME: ultramicroelectrode
WE: working electrode
ZIF: zeolitic imidazolate framework
